# Investigation of the Biosynthetic Potential of Endophytes in Traditional Chinese Anticancer Herbs

**DOI:** 10.1371/journal.pone.0035953

**Published:** 2012-05-22

**Authors:** Kristin I. Miller, Chen Qing, Daniel Man Yuen Sze, Brett A. Neilan

**Affiliations:** 1 Faculty of Pharmacy, The University of Sydney, Sydney, New South Wales, Australia; 2 School of Biotechnology and Biomolecular Sciences, The University of New South Wales, Sydney, New South Wales, Australia; 3 Yunnan Key Laboratory of Pharmacology for Natural Products, School of Pharmaceutical Science, Kunming Medical University, Kunming, China; 4 Department of Health Technology and Informatics, The Hong Kong Polytechnic University, Hung Hom Kowloon, Hong Kong; 5 Australian Centre for Astrobiology, The University of New South Wales, Sydney, New South Wales, Australia; Queen Elizabeth Hospital, Hong Kong

## Abstract

Traditional Chinese medicine encompasses a rich empirical knowledge of the use of plants for the treatment of disease. In addition, the microorganisms associated with medicinal plants are also of interest as the producers of the compounds responsible for the observed plant bioactivity. The present study has pioneered the use of genetic screening to assess the potential of endophytes to synthesize bioactive compounds, as indicated by the presence of non-ribosomal peptide synthetase (NRPS) and polyketide synthase (PKS) genes. The total DNA extracts of 30 traditional Chinese herbs, were screened for functional genes involved in the biosynthesis of bioactive compounds. The four PCR screens were successful in targeting four bacterial PKS, six bacterial NRPS, ten fungal PKS and three fungal NRPS gene fragments. Analysis of the detected endophyte gene fragments afforded consideration of the possible bioactivity of the natural products produced by endophytes in medicinal herbs. This investigation describes a rapid method for the initial screening of medicinal herbs and has highlighted a subset of those plants that host endophytes with biosynthetic potential. These selected plants can be the focus of more comprehensive endophyte isolation and natural product studies.

## Introduction

There is an ongoing need for novel drugs that are highly effective in the treatment of cancer, drug resistant bacteria, fungal infections, emerging viruses and parasitic protozoan infections. Historically, natural products have provided the basis for the majority of new drugs and the bioactive properties of a wide variety of flora is reflected in their continued roles in the traditional healthcare systems of many cultures [1]. The successful use of plants in traditional medicine and modern natural products research has meant that there is a renewed interest in exploiting various aspects of the underlying bioactivities.

Still widely practiced in the modern era, Traditional Chinese Medicine (TCM) can trace its origins back thousands of years, to the dawn of civilisation in China. TCM theory is based on experiences of the effects of the medicines through documented trials to establish knowledge of the use of plants (approximately 5000 species) for the treatment of many diseases [2]. This TCM background has provided the basis for the discovery of several therapeutic agents, including the anticancer agents indirubin [3], camptothecin [4] and harringtonine [5]; aretemisinin (antimalarial) [6] and ephedra (central nervous system stimulant) [7].

Compounds isolated from plant preparations are largely the products of plant metabolism, however, microorganisms living in symbiosis with plants also produce bioactive compounds. Taxol and camptothecin are examples of anticancer compounds synthesized by both plants and endophytes [8], [9]. In their natural environment the internal tissues of plants are frequently colonized by numerous different microorganisms, termed endophytes. Many endophytes produce secondary metabolites which confer major ecological benefits to their host plants including plant growth promotion [10], enhanced resistance to various predators and pathogens [11], [12], and increased drought resistance [13]. Consequently, endophyte derived metabolites may also cause the observed bioactivity and associated beneficial health claims of the TCM host plants [14].

Low molecular weight endophytic secondary metabolites demonstrate a large degree of structural diversity with the largest and most important groups of compounds including the polyketides, amino acid derived compounds, and terpenes [15]. To date genetic methods have been used to screen for biosynthetic pathways involved in secondary metabolism. Genetic screening for microbial natural product genes has largely focused on the detection of the polyketide and non-ribosomal peptide synthesis pathways [16], [17].

Polyketides are produced by many fungi, bacteria, plants and marine organisms [15]. This large family of structurally diverse natural products have already found widespread application as pharmaceuticals including rapamycin (immunosuppressant), erythromycin (antibiotic), lovastatin (anticholesterol drug), and epothilone B (anticancer drug). Polyketides are biosynthesized by large multimodular enzyme complexes termed polyketide synthases (PKSs) which catalyse the polymerisation of acyl-CoA thioester building blocks [18]. PKSs are typically categorized based on their number of subunits (single or multiple) and their mode of synthesis (iterative or modular). The best characterized group of PKSs are the type I PKSs with a single enzyme complex responsible for the biosynthesis of the polyketide backbone. They can be either modular as found in bacteria, or iterative as found in fungi [19]. Extension of the polyketide backbone requires three core PKS domains: an acyltransferase (AT) domain, acyl carrier proteins (ACP) domain and a ketosynthase (KS) domain. The modular structure of type I PKSs and their assembly of polyketides, resembles the organisation of non-ribosomal peptide synthetases (NRPSs) and the production of non-ribosomal peptides.

Complex oligopeptides are formed by NRPSs through the linear condensation of proteinogenic and non-proteinogenic amino acids. NRPSs are involved in the biosynthesis of compounds including penicillin and cephalosporin (antibiotics), cyclosporine A (anti-inflammatory and immunosuppressive activities, and the endophyte-produced fusaricidin (antibiotic). NRPSs are composed of a series of modules, each containing the catalytic units required for the recognition, activation and peptide bond formation of the growing peptide chain by adenylation (A), thiolation (T), and condensation (C) domains, respectively [20]. NRPSs are found in bacteria and fungi feature the three core domains arranged in the order C-A-T [20].

While mechanistically distinct, the highly conserved regions within the KS and A domains are logical targets for the detection of PKS and NRPS biosynthesis genes, respectively. This facilitates the design of degenerate oligonucleotide primers, suitable for genetic screening of secondary metabolism pathways [21], [22], [23].

The location of host plants can affect endophyte populations [24], [25] and for drug discovery potential it is imperative to investigate TCM plants sampled from their natural environment or those cultivated for medicinal purposes. Considering this, plant collections from Yunnan province were selected for two main reasons. Firstly, Yunnan is recognized for its large botanical diversity [26] and secondly, it is a source of many pharmaceutical herbs. TCM herbs with reported anticancer activity were chosen in an effort to increase the likelihood of discovering endophyte produced anticancer compounds [2].

The aim of this study was to assess the use of genetic screening for the identification of plant leads for endophyte natural product investigations. The biosynthetic potential of endophytes was considered via the detection of endophyte PKS and NRPS biosynthesis genes. Total DNA from thirty selected anticancer TCM herbs was genetically screened using four PCR primers sets. This study has identified a subset of TCM herbs found to harbor endophytes with bioactive potential, and these plants can be targeted in future investigations. The use of this genetic screen increases the likelihood of isolating endophytes which biosynthesize novel pharmaceuticals independently of the host plant.

## Methods

### Selection, collection and storage of TCM herbs

Yunnan province is situated in the southwestern region of the People's Republic of China. There is a strong temperature gradient across the province and it contains areas of permanent snow as well as rainforests with temperatures perennially above 10°C [26]. Thirty plant tissue samples (Table 1) were collected during spring in April.2007 from sites within and surrounding Kunming City. No specific permits were required, however, the collections were acknowledged and supported by the Yunnan Provincial government. Potential anticancer TCM herbs were selected based on scientific and traditional literature, their maturity for use as TCMs and their availability for collection at the time of collection. Following collection, samples were placed in sterile bags and stored in the dark at 4°C until processed. Processing was completed as soon as possible following receipt of samples, usually within 24 h.

**Table 1:** Traditional Chinese medicinal herbs collected for genetic screening.

Host Plant Species	Location	Plant Sample	Pharmacological or TCM Activity	Reference
*Aconitum carmichaeli* Debx.	Countryside	Leaf and root	Sedative, analgesic, antiinflammatory, anticancer, toxic	[65]
*Actinidia chinensis* Planch.	Medicinal herb farm	Root and stem	“Heat clearing", antimutagenic, anticancer	[66]
*Agrimonia pilosa* Ledeb. var. japonica (miq.) Nakai	Medicinal herb farm	Above ground	Haemostatic, antiinflammatory, antiinfective	[2]
*Artemisia capillaris* Thunb.	Medicinal herb garden	Leaf and stem	“Heat clearing", antihepatotoxic, antibacterial, antiinflammatory	[2]
*Astilbe rivularis* Buch.-Ham.	Medicinal herb garden	Whole plant	Antiviral	[67]
*Belamcanda chinensis* (L.) DC.	Medical herb farm	Root	Antifungal, antiviral, antiinflammatory, diuretic	[2]
*Bletilla striata* (Thunb.) Reichp.	Medical herb farm	Root/bulb	Haemostatic, anticancer	[2]
*Cephalotaxus fortunei* Hook. F.	Countryside	Leaf and stem	Anticancer	[68]
*Clematis hexapetala* Pall.	Medicinal herb farm	Root	Antibacterial, antifungal	[2]
*Coptis chinensis* Franch.	Medicinal herb farm	Root	Antibacterial, antiinflammatory, affects central nervous system	[2]
*Dendrobium nobile*, Lindl.	Countryside	Stem	Anticancer, antimutagenic	[69]
*Digitalis purpurea* Ehrh.	Medicinal herb garden	Root	Cardiotonic, anticancer	[70]
*Eleutherococcus senticosus* (Rupr. & Maxim) Maxim	Medicinal herb farm	Root	Anticancer, expectorant	[71]
*Leonurus heterophyllus* Sweet	Medicinal herb farm	Above ground	Anticancer, “invigorates blood"	[68]
*Lithospermum erythrorhizon* Sieb.et Zucc.	Medicinal herb farm	Root	Anticancer, antibacterial, antiinflammatory	[2]
*Lonicera japonica* Thunb.	Countryside	Leaf	Antiinfective	[2]
*Lycium chinense* Mill	Medicinal herb garden	Branch and leaf	Anticancer, immune stimulating	[68]
*Pinellia ternata* (Thunb.) Breit.	Countryside	Root	Anti-emetic, mucolytic, anticancer (cervical), discutient	[65]
*Pinellia pedatisecta* Schott	Medicinal herb farm	Root	Toxic, anticancer	[66]
*Paris polyphylla* Smith var. *chinensis* (Franch.) Hara	Medicinal herb farm	Root and leaf	Antiinflammatory, detoxicant, anticancer	[66]
*Rheum palmatum* L.	Medicinal herb farm	Root	Anticancer, detoxicant, antiinflammatory, laxative	[2]
*Rubia cordifolia* L.	Countryside	Leaf and root	Antiinflammatory, antiviral	[72]
*Salvia przewalskii* Maxim	Medicinal herb farm	Root	Anticancer, immune stimulating, antioxidant	[73]
*Salvia yunnanensis* C.H. Wright	Medicinal herb farm	Root	Anticancer, immune stimulating	[73]
*Senecio scandens* Buch.-Ham. Ex D.	Countryside	Leaf and root	Antiinflammatory, anticancer, detoxicant, antibacterial	[68]
*Solanum nigrum* L.	Countryside	Leaf and root	Antimicrobial	[68]
*Sophora davidii* (Franch) Kom. Ex Pavol.	Medicinal herb garden	Leaf and root	Anticancer, antiinflammatory	[74]
*Sophora japonica* L.	Countryside	Leaf and stem	Hemostatic	[68]
*Taxus baccata* L.	Countryside	Leaf and stem	Anticancer	[75]
*Thalictrum foliolosum* DC	Medicinal herb garden	Leaf and root	Anticancer, antiinfective, antihypertensive	[74]

### Surface sterilisation of plant samples

Sections of each plant were carefully washed in running water to remove external soil and debris. Samples were examined to exclude those that showed symptoms of disease or superficial damage. In a method adapted from Cankar et al. [27], plant material was surface sterilized by immersion in 30% hydrogen peroxide for 30 min, followed by rinsing in sterile Milli-Q filtered water (Millipore) and washing with 70% ethanol for 1 min [27]. To confirm that the surface sterilisation process was successful, a section of each sterilized plant was placed into an aliquot of heart infusion broth and incubated at 30°C for three days before examining the media for any growth of plant surface-associated contaminating microorganisms.

### DNA extraction

The different tissue types collected from the one plant were processed together for the total DNA extract. Total genomic DNA was extracted from the surface sterilized plant samples using the Power Plant DNA isolation kit (MoBio Laboratories). Extracted DNA consisted of host plant nuclear and plastid DNA and any endophyte microbial DNA. Extracted DNA was run on an agarose gel and NanoDrop (Thermo Scientific) readings taken to assess concentration and purity. Working stocks of DNA extracts were diluted to 1–10 ng/μL for PCR assays.

### Polymerase Chain Reaction

DNA extracts from TCM plants were screened using PCR primers that targeted various plant, bacterial and fungal genes. The PCR targets and primers employed in this investigation (NRPS, PKS, and the plant RUBISCO genes) are listed in Table 2. Typical gene-specific PCR reactions were carried out in 20 µl reaction volumes that contained 1×reaction buffer (Bioline), 2.5 mM MgCl_2_, 0.2 mM dNTPs (Bioline), 10 pmol each of forward and reverse primers (Sigma-Aldrich, MO, USA), 0.2 U *Taq* DNA polymerase (Bioline) and 1–10 ng of DNA template. Thermal cycling was performed in a MyCycler Thermal Cycler (BioRad), with an initial denaturation at 94°C for 5 min, followed by 30 cycles of 94°C for 30 s, 55°C for 30 s, and 72°C for 1 min, followed by a final extension step at 72°C for 7 min. Degenerate PCRs were also performed in 20 μL reaction volumes containing the same reaction mix as for the non-degenerate primers except that 25 pmol of forward and reverse primers were used and the number of amplification cycles was increased to 35. PCR amplicons were separated by agarose gel electrophoresis in TAE buffer (40 mM Tris-acetate, 1 mM EDTA, pH 7.8), visualized by ethidium bromide staining (0.5 µg /ml) and the GelDoc UV transilluminator (BioRad).

**Table 2:** PCR primers and reaction conditions used in the screening of total DNA extracts from TCM plants.

Target	Primer	Primer Sequence (5′→3′)	Product size (bp)	Altered* reaction conditions	Altered* thermocycling conditions	Reference
Plant RUBISCO enzyme	rbcL-F	tgtcaccacaaacagarackaa	∼1400			[76]
	rbcL-R	caaaattaaatmsgatctctttccatac				
Adenylation domain, Bacterial NRPSs	MTF2	gcnggyggygcntaygtncc	∼1000		94°C for 10 s, 52°C for 30 s, 72°C for 30 s	[77]
	MTR2	ccncgdayttnacytg				
Ketosynthase domain, Bacterial PKSs (Type 1)	DKF	gtgccggtnccrtgngyytc	∼650–700			[41]
	DKR	gcgatggayccncarcarmg				
Adenylation domain, Fungal NRPSs	RJ016-R	arrtcnccngtyttrta	∼300		94°C for 30 s, 50°C for 30 s, 60°C for 1 min; final extension: 60°C for 10 min	[78]
	RJ016-F	tayggnccnacnga				
Ketosynthase domain, Fungal PKSs (Type 1)	LC1	gayccimgittyttyaayatg	∼700	Primers (pmol): LC1, 50; LC2c, 8; LC3, 24; LC5c, 16	94°C for 30 s, 56°C for 10 s, 72°C for 30 s	[79]
	LC2c	gticcigticcrtgcatytc				
	LC3	gcigarcaratggayccica	∼700			
	LC5c	gtigaigticrtgigcytc				
Adenylation domain, Streptomycete NRPSs	A3F	gcstacsysatstacacstcsgg	∼700	10 pmol each primer, 0.25 mM dNTPs, 10% DMSO	94°C for 30 s, 58°C for 1 min, 72°C for 2 min	[33]
	A7R	sasgtcvccsgtscggtas				
Ketosynthase domain, Streptomycete PKS (Type 1)	K1F	tsaagtcsaacatcggbca	∼1200−1400	20 pmol each primer, 0.25 mM dNTPs, 10% DMSO	94°C for 30s, 55°C for min, 72°C for 3 min	[33]
	M6R	cgcaggttscsgtaccagta				

* Altered from standard PCR procedures described.

A spiked PCR targeting the conserved plant RUBISCO gene was used to ensure the absence of PCR inhibitors in the DNA samples. This procedure involved two PCRs per DNA sample, the first containing both sample DNA and an inhibitor free control DNA and the second reaction contained only sample DNA. Lack of amplification in control-spiked reactions was likely to be due to PCR inhibitors in the sample DNA. In this event, DNA was diluted to an extent where the PCR reaction was not inhibited. The second PCR reaction, containing only sample DNA, was used to ensure that DNA was not diluted beyond the PCR detection limits.

### DNA sequencing

PCR amplicons, of the correct size, were purified directly by ethanol precipitation or gel purification using the UltraClean GelSpin DNA Extraction Kit (MoBio Laboratories) in accordance with the manufacturer's instructions.

Amplicons were sequenced using the PRISM BigDye™ cycle sequencing system v3.1 on an ABI PRISM 373 DNA Sequencer (Life Technologies, Carlsbad, USA). PCR products that contained multiple sequences, as observed by multiple peaks in the sequencing chromatograms, were cloned using the TOPO cloning vector (Invitrogen Life Technologies, Carlsbad, USA), transformed into *Escherichia coli* DH5α and a clone library established according to the manufacturer's instructions. Thereafter, colony PCR screening and DNA sequencing of cloned inserts was performed to characterize single amplicons. Sequences were deposited into GenBank.

### Phylogenetic analysis of KS and NRPS fragments

Sequence data were compared to the NCBI dataset on GenBank with the BLASTX algorithm (http://blast.ncbi.nlm.nih.gov/Blast.cgi). The amino acid substrates recognized by the A domain binding pockets were predicted using the NRPSpredictor analysis tool available at (http://www-ab.informatik.uni-tuebingen.de/toolbox/index.php?view=domainpred) [28]. Multiple sequence alignments of KS and A domain protein sequences of PKS or NRPS pathways, respectively, were generated using ClustalX [29] (Figure S1). Gaps and positions with ambiguities were excluded from the phylogenetic analysis. The ProtTest program was used to determine the most appropriate substitution model for the dataset [30]. Phylogenetic analysis was performed using the maximum likelihood methods [31]. Node support values were determined by the approximate Likelihood-Ratio Test (aLRT).

## Results

### Identification of genes encoding bioactivity in TCM plants

The total DNA extracted from thirty TCM plants was expected to contain a mixture of plant and endophyte genes. The DNAs were free from PCR inhibitors, as evidenced by successful PCR amplification of the plant RUBISCO gene. Degenerate PCRs were used to detect putative NRPS and PKS gene sequences originating from bacterial and fungal endophytes in the DNA extracts. Specifically, the PCR screens were successful in targeting endophyte sequences with four streptomycete KS, six streptomycete A, ten fungal KS and three fungal A domain genes detected (Table 3). Some of the amplified PKS and NRPS PCR products could be directly sequenced, however, many contained a mixture of amplicons that needed to be cloned into a TOPO cloning vector. In such cases, at least ten transformants were randomly selected for direct colony PCR and the products sequenced. Amplification of bacterial and fungal A domains and KS domains was confirmed via sequencing and BLASTX (translated) analysis (Table 3). BLASTN (nucleotide) analysis of the PCR products did not reveal any significant matches to the nucleotide database, indicating that the sequences were novel at this level. Endophyte-derived genes were amplified from DNA extracted from different types of plant tissues including the roots, leaves, stems and bark (Table 1).

**Table 3:** PKS and NRPS genes identified with degenerate PCR primers.

Gene	Plant	Sequence identifier	BLASTX Match	Identity (%)	Accession number	Predicted binding pocket (amino acid substrate)^1,2^
Fungal PKS	*Bl. striata*	BS1	Elsinochrome PKS, *Elsinoe fawcettii*	124/161 (77%)	ABU63483	ND
Fungal PKS	*C. fortunei*	CF1	PKS involved in melanin production, *Bipolaris oryzae*	189/210 (90%)	BAD22832	ND
Fungal PKS	*L. heterophyllus*	LH1	non-reduced type PKS for melanin pigment, *Ascochyta rabiei*	232/236 (98%)	ACS74449	ND
Fungal PKS	*L. heterophyllus*	LH2	Uncharacterized PKS, *Botryotinia fuckeliana*	221/221 (100%)	AAR90249	ND
Fungal PKS	*Lo. japonica*	LJ1	Putative non-reduced type PKS, *Penicillium* sp.	193/207 (93%)	ABQ85550	ND
Fungal PKS	*P. ternata*	PT1	Conidial pigment PKS, *Verticillium albo-atrum*	197/211 (93%)	EEY14472	ND
Fungal PKS	*S. scandens*	SS1	PKS 1, *Glarea lozoyensis*	198/220 (90%)	AAN59953	ND
Fungal PKS	*S. scandens*	SS2	Putative melanin PKS, *Penicillium* sp.	205/243 (98%)	ACJ13039	ND
Fungal PKS	*S. scandens*	SS3	PKS 1, *Glarea lozoyensis*	205/243 (84%)	AAN59953	ND
Fungal PKS	*T. baccata*	TB1	Elsinochrome PKS, *Elsinoe fawcettii*	205/243 (84%)	ABU63483	ND
Fungal NRPS	*Pi. pedatisecta*	PPe1	MicC synthetase, *Planktothrix rubescens*	20/34 (58%)	CAQ48260	No prediction
Fungal NRPS	*Pi. pedatisecta*	PPe2	AerB synthetase, *Planktothrix rubescens*	56/95 (58%)	CAQ48266	No prediction
Fungal NRPS	*Pi. pedatisecta*	PPe3	Arthrofactin synthetase, *Burkholderia ghumae*	65/95 (68%)	YP_002908545	No prediction
Bacterial PKS	*D. purpurea*	DL1	Nystatin synthase (NysC) *Streptomyces halstedii*	103/174 (59%)	AAF71776	ND
Bacterial PKS	*Pa. polyphylla*	PPol1	NapC synthase *Streptomyces hygroscopicus*	157/218 (72%)	ABB86421	ND
Bacterial PKS	*Pa. polyphylla*	PPol2	NapC synthase *Streptomyces hygroscopicus*	166/235 (70%)	ABB86421	ND
Bacterial PKS	*Pi. pedatisecta*	PPe4	Arthrofactin synthetase, *Pseudomonas sp*. MIS38	47/63 (74%)	BAC67535	ND
Bacterial NRPS	*D. purpurea*	DL2	Peptide synthetase PhsB, *Streptomyces viridochromogenes*	83/130 (63%)	CAJ14037	DVEHLSLID- (pro)
Bacterial NRPS	*D. purpurea*	DL3	Ansamitocin PKS, *Actinosynnema pretiosum*	121/231 (52%)	AAM54075	-AFALACGM- (val/leu/ile/abu/iva)
Bacterial NRPS	*L. heterophyllus*	LH3	Amino acid adenylation domain *Acidovorax avenae*	18/19 (94%)	YP_972054	DVWNIGLI(thr)
Bacterial NRPS	*L. heterophyllus*	LH4	Pyoverdine synthetase, *Pseudomonas fluorescens*	154/181 (85%)	AAF40219	CVWHFGRI (glu)
Bacterial NRPS	*B. chinensis*	BC1	Amino acid adenylation domain, *Rhodococcus erythropolis*	192/195 (98%)	ZP_04387369	DATFAGGI (leu/ile/val)
Bacterial NRPS	*B. chinensis*	BC2	Amino acid adenylation domain, *Rhodococcus erythropolis*	224/228 (98%)	ZP_04387369	DATFAGGI (leu/ile/val)

1 In silico prediction of the amino acid substrate recognized by putative NRPS fragments [28].

2 ND =  not done.

Ten unique fungal KS domain sequences (∼700 bp) were amplified from total DNA extracts of *Bl. striata*, *C. fortunei*, *L. heterophyllus*, *Lo. japonica*, *P. ternata*, *S. scandens,* and *T. baccata* (Table 3). The amplified sequences possessed between 76% and 100% amino acid identity to known fungal PKSs. Specifically, sequence LH2 shared 100% sequence identity with an uncharacterized PKS from *Botryotinia fuckeliana.* Other fungal PKS amplicons were similar to genes involved in the production of polyketides, such as melanin and the toxic pigment elsinochrome.

Screening of total TCM plant DNA successfully amplified ∼300 bp fragments of the fungal NRPS A domain. Of all the DNAs screened only DNA from *Pi. pedatisecta* produced NRPS gene fragments (PPe1, PPe2, PPe3). BLASTX analysis revealed that the predicted translated DNA sequences were homologous to bacterial NRPSs, with sequence similarity between 58% and 68%. Prediction of the eight amino acids that form the A domain active site, and the amino acids that bind to the active residues, was not successful possibly indicating that the algorithm is not sufficiently robust to predict fungal NRPS module specificity [32].

Bacterial directed primers were also used to screen for PKS and NRPS genes [33]. Degenerate primers targeting the genes coding for the KS domain of actinobacterial PKSs amplified a total of four unique amplicons from the plant DNA extracts, including *D. purpurea* (one), *P. polyphylla* (two), and *P. peditasecta* (one). Predicted translation of the gene fragments was afforded through the BLAST program and subsequent peptide sequence analysis established that three of the fragments were similar to sequences of PKSs involved in the biosynthesis of the antifungal compound nystatin (DL1, 59%) and the anticancer and antibiotic compound geldanamycin (PPo1, 72% and PPo2, 70%). The sequence of PPe4 was shorter (∼235 bp) and had 74% similarity to arthrofactin synthetase from *Pseudomonas* sp. MIS38 (Table 3).

Screening of the TCM plant DNA extracts for bacterial NRPS A domain gene sequences revealed six unique amplicons from the extracts of *D. purpurea*, *L. heterophyllus* and *B. chinense*. The translated sequences of five of these amplicons showed high sequence similarity, 57–98%, to bacterial NRPS synthetases (Table 3). Importantly, amplicons BC1 and BC2 showed significant similarity (98%) to an adenylation domain of an uncharacterized *Rhodococcus erythropolis* NRPS. Of relevance to drug discovery, the product amplified from *D. purpurea* (DL3) had 65% sequence similarity to the ansamitocin synthase from *Actinosynnema pretiosum*. Unlike the PKS amplicons, which were all similar to published *Streptomyces* sp. sequences, the NRPS PCR amplicons were similar to sequences of diverse bacteria, including the Proteobacteria and different genera of the Actinobacteria group (Table 3).

The deduced translated A domains sequences were analysed to determine if they contained an eight residue binding pocket, and the amino acid that was likely to be bound by the pocket. Predictions were successful for the bacterial NRPS sequences including fragments BC1 and BC2 that are likely to possess the same amino acid binding pocket, and could bind either a Leu, Ile or Val residue (Table 3). Analysis of the two fragments detected from the endophytes of *L. heterophyllus*, LH3 and LH4, were shown to possess different binding pockets and thus would be involved in the addition of different amino acids to the growing peptide chain produced by the NRPS (Table 3).

### Phylogenetic distribution of PKS and NRPS genes from TCM endophytes

**Figure 1:**
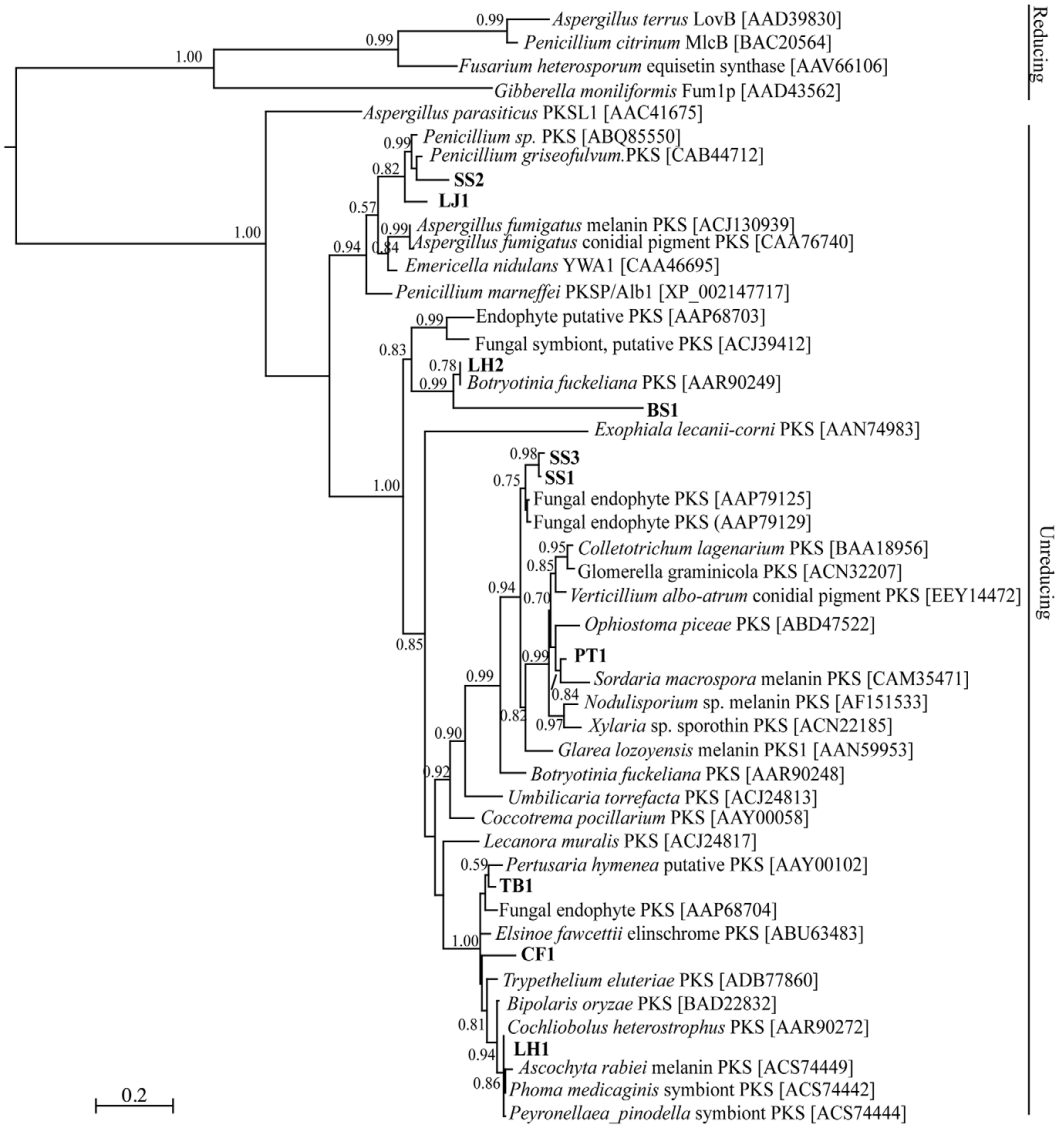
Phylogenetic analysis of putative fungal type I PKSs in TCM herbs. Evolutionary relationships were determined by maximum likelihood analysis using the LG substitution model. Branch lengths indicate inferred divergence of amino acid sequences. Numbers adjacent to the nodes indicate aLRT support, with support values >50% considered to be significant. Accession numbers for sequences obtained from GenBank are indicated. The scale bar represents 0.2 amino acid changes.

In an attempt to investigate the homology and novelty of amplicons from the genetic screen, the deduced amino acid sequence of the KS and A domain gene fragments were used in multiple sequence alignments and the phylogenies reconstructed. The unrooted phylogenetic tree of fungal PKSs (Figure 1) revealed the presence of two main clades that correspond to the two largest structural classes of fungal polyketides: reduced and unreduced. All fungal KS domain sequences amplified in this study belonged to the clade of PKSs which synthesize unreduced PKs. The proteins were closely grouped to PKSs involved in the biosynthesis pathways of various pigments including melanin (LH1, PT1), other pigments from *Penicillium* sp. and *Elsinoe fawcetti* (CF1, LJ1, SS2), or putative PKSs derived from endophytes and other fungal symbionts (BS1, LH2, SS1, SS3, PT1). The gene products of SS1 and SS3 formed a highly supported clade and are possible homologs of the same biosynthetic pathway.

**Figure 2:**
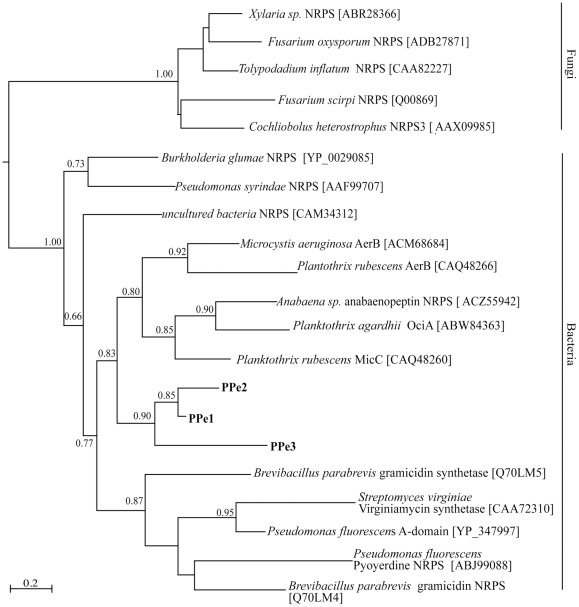
Phylogeny of fungal NRPSs. Relationships inferred by maximum likelihood analysis of fungal NRPS adenylation domain fragments using the WAG substitution model. Branch lengths indicate inferred divergence of amino acid sequences. Numbers adjacent to nodes indicate aLRT support, with support values >50% considered significant. Bacterial and fungal reference sequences are included in the analysis and the accession numbers for these sequences obtained from GenBank are indicated. The scale bar represents 0.2 amino acid changes.

Phylogenetic analysis was performed on the inferred amino acid sequences of NRPS A domains (Figure 2). The three NRPS A domains sequences identified in this study were amplified from the extract of *Pi. pedatisecta* and although each sequence was unique, the sequences (PPe1, PPe2 and PPe3) were clustered in the phylogenetic tree indicating that they most probably have a common phylogenetic ancestor and/or similar function. This cluster of fragments also grouped with cyanobacterial NRPSs. Related NRPSs included those involved in the synthesis of a range of bioactive compounds; MicC in the synthesis of the pentapeptide microginin in *Planktothrix rubescens* [34], AerB in the synthesis of the aeruginosin protease inhibitors in *Microcystis viridis* [35], and OciA in the synthesis of cyanopeptolin [36]. Despite the fact that fungal specific PCR primers were used, these A domain sequences had greater similarity to NRPSs of bacterial origin with bacterial and fungal NRPSs clustered separately in the phylogenetic tree (Figure 2).

**Figure 3:**
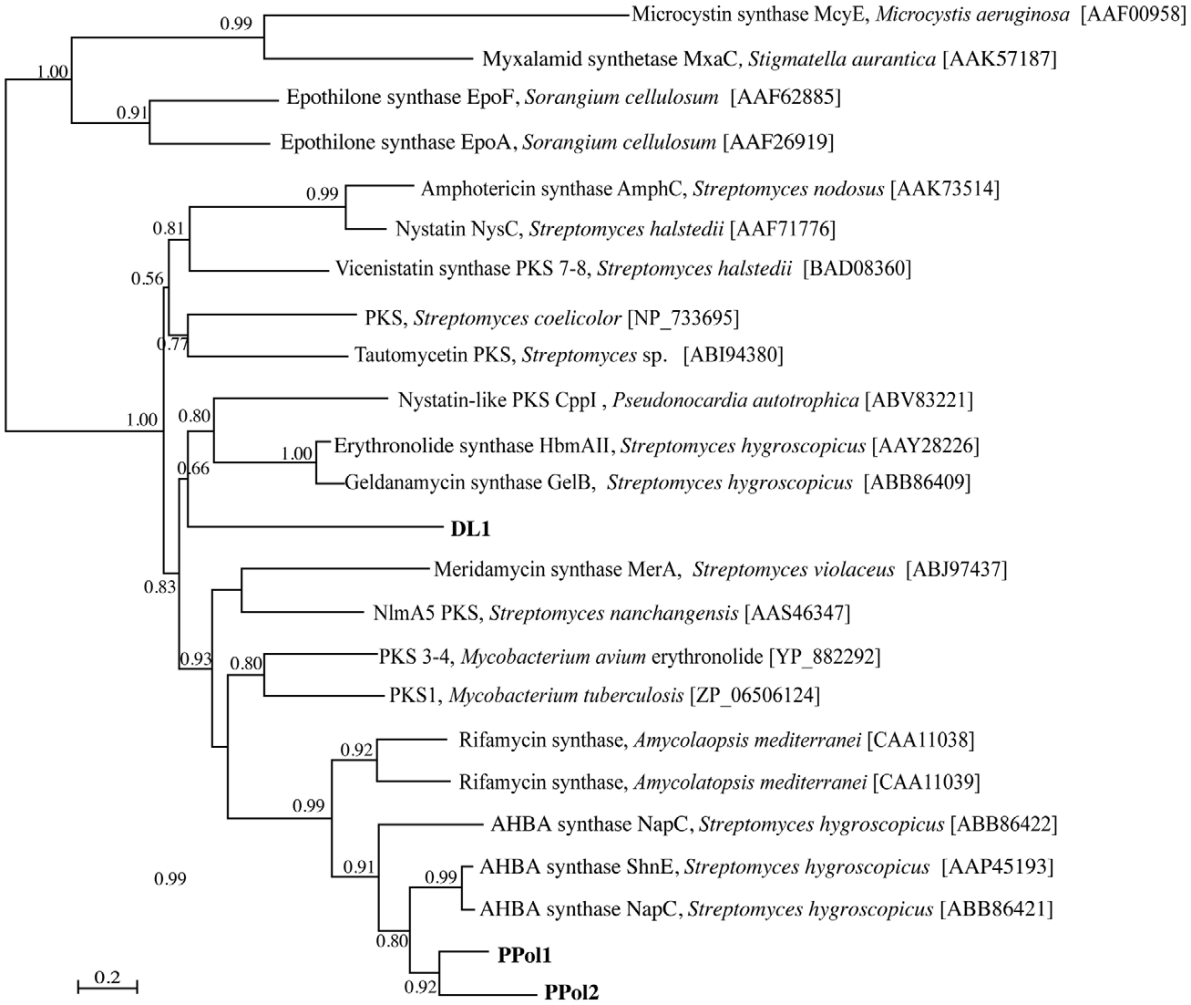
Phylogeny of bacterial type I PKSs. Relationships inferred by maximum likelihood analysis of bacterial PKS ketosynthase domain fragments, using the LG substitution model. Branch length indicates inferred divergence of amino acid sequences and numbers adjacent to the nodes indicate aLRT support, with support values >50% considered significant. The accession numbers for all sequences obtained from GenBank are indicated. The scale bar represents 0.2 amino acid changes.

Phylogenetic analysis of inferred bacterial KS domains revealed that the endophyte sequences clustered with KS domains of Actinobacteria (Figure 3). Sequences PPol1 and PPol2 were closely related to each other and also clustered with PKS fragments involved in the biosynthesis of the anticancer antibiotic geldanamycin, from *Streptomyces hygroscopicus* [37]. The DL1 sequence clustered within a clade containing KS domains, involved in the tailoring of geldanamycin and herbimycin in *S. hygroscopicus,* and an uncharacterized KS domain from *Pseudonocardia autotrophica*. The sequence PPe4 was not included in the bacterial PKS multiple sequence alignment and phylogenetic tree, because it was significantly shorter than the other fragments (∼80 amino acids).

**Figure 4:**
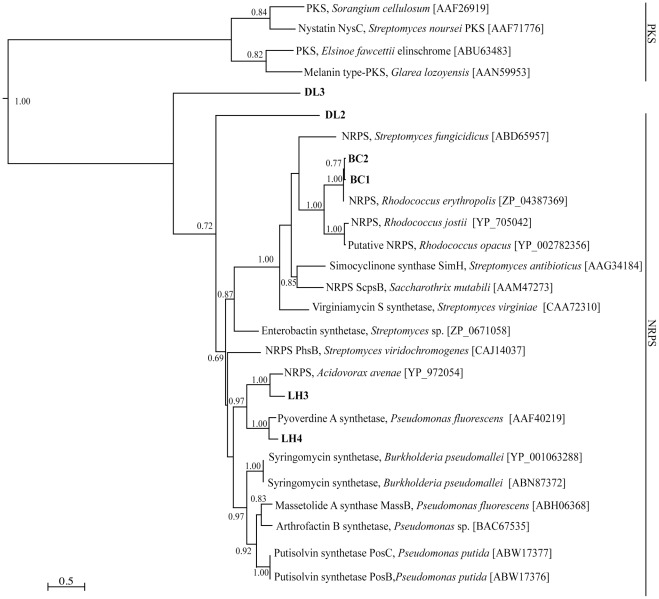
Phylogeny of bacterial NRPS sequences. Relationships inferred by maximum likelihood analysis of bacterial NRPS adenylation domain fragments using the LG substitution model. Branch lengths indicate inferred divergence of amino acid sequences. Numbers adjacent to the nodes indicate aLRT support, with support values >50% considered significant. The accession numbers for all sequences obtained from GenBank are indicated. The scale bar represents 0.5 amino acid changes.

The phylogenetic tree containing bacterial NRPS A domain sequences demonstrated two main clusters from the Actinobacteria and Proteobacteria (Figure 4). Sequences BC1 and BC2 were closely related and clustered with an uncharacterized NRPS biosynthesis pathway from *Rhodococcus erythropolis*. Although the function of the *R. erythropolis* NRPS is unknown, it also grouped with A domain sequences from other Actinobacteria. Sequences LH3 and LH4 were related to NRPS sequences from the Proteobacteria, specifically *Pseudomonas fluorescens* and *Acidovorax avenae*, respectively. The sequences DL3 grouped with the clade of bacterial A domain sequences, however, had low homology to known NRPSs.

## Discussion

Natural product drug discovery investigations are supported by a vast number of candidate systems and environments available for study, presenting the necessity to optimise the targets chosen for investigation. Ethnopharmocological approaches have been affirmed by the discovery of novel bioactive compounds from traditional medicinal plants [3], [6], [38]. Additionally endophytes are capable of producing a wide variety of natural products, contributing to the overall bioactivity of the host plant. Thus, they are considered to be a valuable source of novel bioactive compounds and an avenue to increase the breadth of natural product studies [39]. The present study took advantage of the immense knowledge of TCM plants to focus on plants of pharmaceutical interest. This preliminary study screened the metabolic potential of endophytes using PKS and NRPS genes as a proxy for endophytic production of bioactive compounds. The use of these genes reflects the noted involvement of PKSs and NRPSs in the production of many natural products [21], [40], [41]. This screen has the potential to be applied to the endophytes of other TCM plant species or growth climates.

PKS and NRPS genes are appropriate targets for detection of small molecule biosynthesis systems [21], [40], [41]. Genetic screening results demonstrated the presence of at least one NRPS/PKS biosynthetic pathway in 36% (11/30) of the plant DNA extracts. Furthermore, PKS genes were detected more frequently in the total DNA samples (30%, 9/30) than NRPS genes (13%, 4/30). PKS and NRPS biosynthesis genes have previously been detected in fungal and bacterial isolates from ecological niches similar to those inhabited by endophytes. Fungal and bacterial isolates of sponges (18% and 13.8%, respectively) and marine (90%) and freshwater (65%) cyanobacteria were positive for PKS and NRPS genetic screens [23].

Due to the fact that plants are host to diverse endophyte populations, it was speculated that many of the total DNA extracts would contain PKS and NRPS genes. However, genetic screening for endophyte biosynthesis genes has not previously been attempted using total plant DNA extracts. In this study, DNA samples often contained only one PKS/NRPS sequence, inferring that there may be few biosynthesis genes present in the endophytes of the sampled plants. It is probable that only a few endophytes biosynthesize compounds that increase plant viability, and these endophytes may dominate the population, and any PCR results. Additionally, many samples failed to reveal any endophyte PKS or NRPS genes. This indicates that either endophytes within the samples did not contain PKS or NRPS biosynthesis genes or that the biosynthesis gene sequences were too divergent to be amplified by the reaction described. Alternatively, endophytes involved in the biosynthesis of bioactive compounds in these plants may have been absent from the tissue type used for the DNA extractions [42]. Instead endophytes could inhabit an alternate host tissue and export bioactive compounds for storage in the tissue used medicinally [43].

Actinobacteria, in particular *Streptomyces* sp., are significant secondary metabolite producers, synthesising a functionally diverse class of bioactive compounds, often through PKS and NRPS pathways [44], [45]. Although Actinobacteria are only a subset of the total bacterial population, sequences related to non-actinobacteria NRPS genes were also detected using the actinobacterial primers (Figure 4). This is possible, due to the conserved gene regions within the KS and A domains [46]. However, the type I PKS and NRPS primers used in this study cannot amplify all PKS or NRPS genes, suggesting that these primers underestimate endophytes' KS and NRPS gene diversity within the plants. This limitation is in part due to the absence of divergent sequences in the sequence databases and primers which are not designed to detect atypical PKS or NRPS genes. Consequently, some interesting pathways may be overlooked with this approach. Sequence-based approaches will improve from advances in our understanding of the molecular genetics of PKS and NRPS biosynthesis.

Phylogenetic analysis of putative fungal KS domains showed that sequences clustered into two main groups (Figure 1). PKSs which synthesize partially reduced polyketides, such as lovastatin and T-toxin, were clustered separately from those PKSs that synthesize unreduced polyketides, examples of which include aflatoxin and melanin. This division is supported by previous phylogenetic investigations of fungal PKSs [47], [48]. All ten sequences from the TCM samples were within the cluster of PKSs that synthesize unreduced polyketides, specifically, pigments. Fungal pigments show a range of bioactivities such as elsinchrome, which possesses phytotoxic activities [49], and the napthopyrones, which possess antimicrobial activities [50], [51]. It can be concluded that the detection of pigment-type biosynthesis genes indicates the biosynthetic potential of TCM endophytes. The putative fungal KS domain fragments identified; BS1, CF1, LH1, LH2, SS1, SS3 and TB1, showed close relationships with KS domains of symbiotic fungi, including endophytes and mycobionts of lichens (Figure 1). These amplified KS domains may encode biosynthetic pathways unique to symbiotic fungi and thus be involved in the host-symbiont relationship.

Phylogenetic analysis of the fungal A domains demonstrated that the sequences were clustered with NRPSs from bacteria (Figure 2). Some sequences showed similarity to bacterial PKS sequences involved in the production of cyclic lipopeptides, such as arthrofactin synthetases (Table 3). Cyclic lipopeptide production by *Pseudomonas fluorescens* was shown to play a key role in biofilm formation, motility and the antifungal activity of strain SBW25. Thus the detected fungal A domains could be involved in the biosynthesis of novel fungal peptides. The amplified A domains were related to NRPS pathways involved in the synthesis of compounds with a range of bioactivities; microginin, an angiotensin-converting enzyme inhibitor [52], anabaenopeptin, a serine protease inhibitor [53], and aeruginosin, a thrombin inhibitor [54]. Protease inhibitor-type compounds are significant because of the biochemical processes they control. For example serine protease inhibitors possess cell cytotoxic activities and are used for the treatment of a wide variety of human diseases [55].

Sequences identified from the bacterial NRPS screen were phylogenetically grouped with diverse bacterial NRPS biosynthesis pathways. The sequences LH3 and LH4 were grouped with proteobacterial NRPSs involved in the synthesis of the siderophores pyoverdine and enterobactin [56], [57]; antibiotics, massetolide A and virginiamycin [58], [59]; and the biosurfactant, arthrofactin [60]. It appears that these putative A domain genes could be involved in the synthesis of novel and biologically active peptides, which in turn add to the medicinal activity of the host plants. Gene fragments BC1 and BC2 were related to an A domain of an uncharacterized NRPS pathway of *Rhodococcus erythropolis*. While this specific NRPS gene is uncharacterized, *Rhodococcus* spp. are known to contain a large set of NRPS and PKS genes with their biotechnological significance arising from their existence in harsh environmental niches [61], [62]. It is proposed that the products of this NRPS may assist endophytes to survive inside the plant. The putative A domain sequence DL3 failed to cluster with known NRPSs and therefore may represent divergent NRPSs, for the production of unique peptide-derived compounds.

Of interest in the phylogenetic analysis of bacterial PKS fragments, is that fragments PPol1 and PPol2 grouped with various enzymes of *Streptomyces hygroscopicus* involved in geldanamycin biosynthesis. This suggests that the endophyte genes could encode pathways for the production of similar anticancer antibiotics [63]. The detection of endophyte associated biosynthesis genes in *P. polyphylla* highlights the fact that this plant is a good candidate for further endophyte investigations involved in isolation endophyte and characterisation of their associated bioactive compounds. Endophytes offer sustainable production of plant associated natural products, and have undeniable application in the development of compounds in drug discovery [64].

In summary, TCM herbs are host to diverse bacteria and fungi with the potential to synthesize secondary metabolites which would contribute to the plant's chemical composition. In this particular study, genetic screening revealed endophyte-derive PKS and NRPS fragments with putative roles in the biosynthesis of secondary metabolites with a wide array of biological activities. This screening allows further investigations to focus on plants that host endophytes with the greatest potential to produce polykletide and nonribosomal peptide-based bioactive substances. To further investigate links between PKS/NRPS genes and bioactivity, elucidation of the full biosynthetic pathways is required from endophytes. Future studies of the plant candidates may lead to harnessing endophytes, their genetic resources, and associated compounds which are valuable leads for drug discovery investigations, as well as understanding how endophytes contribute to the bioactivity of medicinal plants. In addition, it remains to be determined whether the PKS and NRPS genes identified in this study are functionally dedicated to specialized endophyte-based activities.

## Supporting Information

Figure S1 **Multiple sequence alignments.** The multiple sequence alignments of KS domains protein sequences, from PKS pathways, and A domain protein sequences, from NRPS pathways, were generated using ClustalX.
(DOC)
