# Supplementary material for: Investigation of the Biosynthetic Potential of Endophytes in Traditional Chinese Anticancer Herbs
Source: PLoS One. 2012 May 22;7(5):e35953. doi: 10.1371/journal.pone.0035953 (PMC3358349; doi:10.1371/journal.pone.0035953)
Supplement: Figure S1 — Multiple sequence alignments. The multiple sequence alignments of KS domains protein sequences, from PKS pathways, and A domain protein sequences, from NRPS pathways, were generated using ClustalX. (DOC) [file pone.0035953.s001.doc]

Figure S1:

Fungal PKS multiple sequence alignment

ABQ85550 ALQADPAQRLALLTAYEAFEMAGFIPDSTPSTQKNRVGVFYGMTSDDYR--EINSGQDID

CAB44712 ALQADPAQRLALLTAYEALEMAGFIPDSTPSTQKNRVGVFYGMTSDDYR--EVNSGQDID

**SS2 ALQADPAQRLALLTAYEALEMAGFIPDSTPSTQKNRVGVFYGMTSDDYR--EVNSGQDID**

**LJ1 ALQADPAQRMALLTAYEAFEMVGFIPDSTPSTQKNRVGVFYGMTSDDYR--EINSGQDID**

ACJ13039 ALQADPAQRLALLSAYEALEMAGFVPNSSPSTQRDRVGIFMGMTSDDYR--EINSGQDID

CAA76740 ALQADPAQRLALLSAYEALEMAGFVPNSSPSTQRDRVGIFMGMTSDDYR--EINSGQDID

CAA46695 ALQADPAQRLALLTAYEALEGAGFVPDSTPSTQRDRVGIFYGMTSDDYR--EVNSGQDID

XP_002147717 ALQADPGQRLALVTAYEALEMAGFVPDSTPSTQKDRVGIFYGMTSDDYR--EINSGQDID

AAC41675 APQMDPAQRMALMSTYEAMERAGLVPDTTPSTQRDRIGVFHGVTSNDWM--ETNTAQNID

ACS74449 AAQTDPMGRLALTTAFEALEMSGYVPNRTPSTKLNRIGTFYGQTSDDWR--EINAAQNID

ACS74442 AAQTDPMGRLALTTAFEALEMSGYVPNRTPSTKLNRIGTFYGQTSDDWR--EINAAENID

ACS74444 AAQTDPMGRLALTTAFEALEMSGYVPNRTPSTKLNRIGTFYGQTSDDWR--EINAAENID

**LH1 AAQTDPMGRLALTTAFEALEMSGYVPNRTPSTKLNRIGTFYGQTSDDWR--EINAAENID**

BAD22832 AAQTDPMGRLALTTAYEALEMSGYVPNRTPSTKLERIGTFYGQTSDDWR--EINAAENID

AAR90272 AAQTDPMGRLALTTAYEALEMSGYVPNRTPSTKLERIGTFYGQTSDDWR--EINAAENID

ADB77860 AAQTDPMGRLALTTAYEALEMSGYVPNRTPSSKLERIGTFYGQTSDDWR--EINAAENVD

AAY00102 AAQTDPMGRLALTTAYEALEMSGYVPNRTPSTKLHRIGTFYGQTSDDWR--EINAAENVD

**TB1 AAQTDPMGRLALVTAYEALEMSGYVPNRTPSTKLHRIGTFYGQTSDDWR--EINAAENVD**

AAP68704 AAQTDPMGRLALVTAYEALEMSGYVPNRTPSTKLHRIGTFYGQTSDDWR--EINAAENVD

ABU63483 AAQTDPMGRLALVTAYEALEESGYVPNRTPSTKLHRIGTFYGQTSDDWR--EINAAENVD

CF1 AAQTDPMGRLALVTAYEALEMSGYVPGRTPSTRNHRVGTFYGQTSDDWR--EINAAENVD

ACJ24817 AAQTDPMQRLALVTAYEALEMAGYVPNRTASTKLNRIGTFYGQTSDDWR--EVNAAQHID

PT1 AFQTDPMQRMALTTAYEALEMSGYVPNRTPSTRLDRIGTFYGQTSDDWR--EINAAQEVD

CAM35471 AFQTDPMQRMALTTAYEALEMSGYVPNRTPSTKLDRIGTFYGQTSDDWR--EINAAQEVD

BAA18956 AFQTDPMQRMALTTAYEALEMCGYVPNRTPSTRLDRIGTFYGQTSDDWR--EINAAQEVD

ACN32207 AFQTDPMQRMALTTAYEALEMCGYVPNRTPSTKLDRIGTFYGQTSDDWR--EINAAQEVD

EEY14472 AYQTDPMQRMALTTAYEALEMSGYVPNRTASTRLDRIGTFYGQTSDDWR--EINAAQEVD

ABD47522 ALQTDPMQRMAISTAFEALEMSGYVPNRTPSTRLDRIGTFYGQTSDDWR--EINAAQSVD

AF151533 AFQTDPMQRLALTTAYEALEMSGYVPNRTPSTRLDRIGTFYGQTSDDWR--EINAAQEVD

ACN22185 AFQTDPMQRMALTTAYEALEMSGYVPNRTPSTRLDRIGTFYGQTSDDWR--EINAAQEVD

SS3 AFQTDPMQRMALSTAYEALEMSGYIPNRTRSTKLDRIGTFYGQTSDDWR--EINAAQEID

SS1 AFQTDPMQRMALSTAYEALEMSGYIPNRTRSTKLDRIGTFYGQTSDDWR--EINAAQEID

AAP79125 AFQTDPMQRMALSTAYEALEMSGYVPNRTRSTQLDRIGTFYGQTSDDWR--EINAAQEVD

AAP79129 AFQTDPMQRMALSTAYEALEMSGYVPNRTRSTKLDRIGTFYGQTSDDWR--EINAAQEVD

AAN59953 AFQTDPMQRMALSTAFEALEMSGYTPNRTPSTRLDRVGTFYGQTSDDWR--EINAAQEVD

AAR90248 AYQTCPMQRLGLATAYEALEMAGYVPNRTHSTKLDRIGTFYGQTSDDWR--EINAAQDVD

ACJ24813 AVQTDPMQRLSLVTAYEALEMAGYVPNRTPSTKLDRVGTFYGQTSDDWR--EINAAQDID

AAY00058 ATQTDPMQRLALITAFEALEMSGYVPNRTPSTKLDRIGTFYGQTSDDWR--EINESQDID

LH2 AMQTDPMQRLAISTAYEAMEMSGFVRDRTPSTQAHRIGTFYGQTSDDWR--EINAAQDID

AAR90249 AMQTDPMQRLAISTAYEAMEMSGFVRDRTPSTQAHRIGTFYGQTSDDWR--EINAAQDID

AAP68703 AAQTDPMQRLMLVTAYEAMEMGGIVPGRTPSTKRDRIGTFYGQTSDDWR--EINAAQDID

ACJ39412 AAQTDPMQRLMLVTAYEAMEMSGFVPDRTPSTKRDRIGTFYGQTSDDWR--EINAAQDID

AAN74983 AEQTDPQQRLALVTAHEALEMSGFVPNRTPSTQLDRIGTYYGQTGDDYR--EWNASQDVQ

BS1 GWLCERSDTINQSSSHRDLLR---TDQRLARNQCRPRHYLLCKLVVVIR--LITAN----

AAD39830 AESMDPQHRLLLETVYEAVTNAGMRIQDLQGTS---TAVYVGVMTHDYETVSTRDLESIP

BAC20564 AETIDPQQRLLLETVYEAVSNAGLRIQGLQGSS---TAVYVGMMTHDYETIVTRELDSIP

AAV66106 AEAIDPQQRLLLETVYESLCAAGLPIERLRGSD---TGVYIGLMCDDWSSMIQKDIDALP

AAD43562 VSDIDPQQRMLLEVAYECMQSSGQ--TNWRGSN---IGCYVGVWGEDWLDLHSKDLYDSG

: .. . .

ABQ85550 TYFIPGGNRAFTPGRINYYFKFSGPSVSVDTACSSSLAAIHVACNSLWRNECDSAVAGGV

CAB44712 TYFIPGGNRAFTPGRINYYFKFSGPSVSVDTACSSSLAAIHVACNSLWRNESDSAVAGGV

SS2 **TYFIPGGNRAFTPGRINYYFKFSGPSVSVDTACSSSLAAIHVACNSLWRNECDSAVAGGV**

LJ1 **TYFIPGGNRAFTPGRINYYFKFSGPSVSVDTACSSSLAAIHVACNSLWKNDCDSAVAGGV**

ACJ13039 TYFIPGGNRAFTPGRINYYFKFSGPSVSVDTACSSSLAAIHLACNAIWRNDCDTAISGGV

CAA76740 TYFIPGGNRAFTPGRINYYFKFSGPSVSVDTACSSSLAAIHLACNAIWRNDCDTAISGGV

CAA46695 TYFIPGGNRAFTPGRINYYFKFSGPSVSVDTACSSSLAAIHLACNSIWRNDCDTAITGGV

XP_002147717 TYFIPGGNRAFTPGRINYHFKFSGPSVSVDTACSSSLSAIHMACNALWKNDCDTAIAGGT

AAC41675 TYFITGGNRGFIPGRINFCFEFAGPSYTNDTACSSSLAAIHLACNSLWRGDCDTAVAGGT

ACS74449 TYFITGGVRAFAPGRINYYFKFSGPSYSIDTACSSSLAAIQLACTSLWAGDCDTACAGGL

ACS74442 TYFITGGVRAFAPGRINYYFKFSGPSYSIDTACSSSLAAIQLACTSLWAGDCDTACAGGL

ACS74444 TYFITGGVRAFAPGRINYYFKFSGPSYSIDTACSSSLAAIQLACTSLWAGDCDTACAGGL

LH1 **TYFITGGVRAFAPGRINYYFKFSGPSYSIDTACSSSLAAIQLACTSLWAGDCDTACAGGL**

BAD22832 TYFITGGVRAFAPGRINYYFRFSGPSYSVDTACSSSLAAIQLACTSLWAGDCDTACAGGL

AAR90272 TYFITGGVRAFAPGRINYYFKFSGPSYSVDTACSSSLAAIQLACTSLWAGDCDTACAGGL

ADB77860 TYFITGGVRAFAPGRINYYFKFSGPSFSIDTACSSSLAAIQLACTSLWAGDCDTACAGGL

AAY00102 TYFITGGVRAFAPGRINYYFKFSGPSYSIDTACSSSLAAVQLACTSLWAGDCDTACAGGL

TB1 **TYFITGGVRAFAPGRINYYFKFSGPSYSVDTACSSSLAAIQLACTSLWSGDCDTACAGGL**

AAP68704 TYFITGGVRAFAPGRINYYFKFSGPSYSVDTACSSSLAAIQLACTSLWAGDCDTAIAGGL

ABU63483 TYFITGGVRAFAPGRINYYFKFSGPSFSIDTACSSSLAAIQLACTSLWAGDCDTACAGGL

CF1 TYFITGGVRAFAPGRINYYFKFSGPSFSVDTACSSSLSAIQLACTSLWAGDLDTACAGGL

ACJ24817 TYFIPGGVRAFAPGRIDYHFKFSGPSFSIGTACSSSLAAIQLACTSLQAGDCDTAVAGGV

PT1 TYFITGGVRAFGPGRINYHFGFSGPSLNIDTACSSSAAAMQVACTALWARDCDTAIVGGL

CAM35471 TYFITGGVRAFGPGRINYHFGFSGPSLNIDTACSSSAAALQVASTALWARDCDTAIVGGL

BAA18956 TYYITGGVRAFGPGRINYHFGFSGPSLNVDTACSSSAAALNVACNSLWQKDCDTAIVGGL

ACN32207 TYYITGGVRAFGPGRINYHFGFSGPSLNIDTACSSSAAALNVACNALWVKDCDTAIVGGL

EEY14472 TYFITGGVRAFGPGRINYHFGFSGPSLNIDTACSSSAAALQIACTSLWAKDCDTAVVGGL

ABD47522 TYYITGGVRAFGPGRINYHFGFSGPSLNIDTACSSSAAAMNVACTSLWARDCDTAIVGGL

AF151533 TYYITGGVRAFGPGRINYHFGFSGPSLNIDTACSSSAAALQVACTSLRAKECDTAIVGGL

ACN22185 TYFITGGVRAFGPGRINYYFGFSGPSLNIDTACSSSAAAMHVACTSLWAKECDTAIVGGL

SS3 TYFITGGVRAFGPGRINYHFGFSGPSFNIDTACSSSAAAMQLACTSLVARECGTAIVGGL

SS1 TYFITGGVRAFGPGRINYHFGFSGPSFNIDTACSSSAAAMQLACTSLVARECDTAIVGGL

AAP79125 TYFITGGVRAFGPGRINYHFGFSGPSFNIDTACSSSAAAMQLACTSLWAGDCDTAIVGGL

AAP79129 TYFITGGVRAFGPGRINYHFGFSGPSFNIDTACSSSAAAMQLACTSLWAGDCDTAIVGGL

AAN59953 TYFITGGVRAFGPGRINYHFGFSGPSFNVDTACSSSAAALQLAYTSLCAKDCDTAIVGGL

AAR90248 TYFITGGVRAFGPGRINYHFGFSGPSFNIDTACSSSAAALQLACTSLWAGDCDTAVVGGL

ACJ24813 TYFITGGVRAFGPGRINYHFKFSGPSFSVDTACSSGMAAIQLACTSLWSGNCDTAIAGGV

AAY00058 TYFITGGVRAFGPVSINYHFKFSGPSFSVDTACSSSMAAIQLACTSLWAGDCDTAVAGGM

LH2 TYFITGGVRAFGPGRINYHFGFSGPSYSVDTACSSSMAAINLAVTSLRAGDCDTVFAGGM

AAR90249 TYFITGGVRAFGPGRINYHFGFSGPSYSVDTACSSSMAAINLAVTSLRAGDCDTVFAGGM

AAP68703 TYFISGGVRAFGPGRLNYFFKWSGPSFSVDTACSSSFAALNIACTALRAGECDTAFSGGA

ACJ39412 TYFISGGVRAFGPGRINYFFKFSGPSFSVDTACSSSMAALNIAVTSLRAGECDTAFTGGA

AAN74983 TYYISGNDRAFGPGRINHHFKFGGPSMSIDTACSSSAVALNVACTALWANDCATAVVGGM

BS1 IIQISGGVRAFGPGRINYHFGFSGPSFSIDTACSSSFAAIQLACTSLRAGECDTVFTGGM

AAD39830 TYSATGVAVSVASNRISYFFDWHGPSMTIDTACSSSLVAVHLAVQQLRTGQSSMAIAAGA

BAC20564 TYSATGVAVSVASNRVSYFFDWHGPSMTIDTACSSSLAAVHLAVQQLRTGESTMAVAAGA

AAV66106 TYTGTGTARSILSNRVSYFFDWQGPSMTIDTACSSSLVAVHEAVRLLRSGDSTVAIAAGA

AAD43562 TYRVSGGHDFAISNRISYEYDLKGPSFTIKAGCSSSLIALHEAVRAIRAGDCDGAIVAGT

.* . :.. : *** . :.***. *:: * : : . .*

ABQ85550 NILTNPDNHAGLDRGHFLSRTGNCTTFDDGADGYCRADGIGSVVLERLEDAQADNDPIYG

CAB44712 NILTNPDNHAGLDRGHFLSRTGNCTTFDDGADGYCRADGIGSVVLKRLEDAQADNDPIYG

SS2 **NILTNPDNHAGLDRGHFLSRTGNCTTFDDGADGYCRADGIGSIVIKRLEDAQADNDPIYG**

LJ1 **NVLTNPDNHAGLDRGHFLSRGGNCNTFDDGADGYCRADGIGSVVLKRLEDAQADNDPIYG**

ACJ13039 NLLTNPDNHAGLDRGHFLSRTGNCNTFDDGADGYCRADGVGTIVLKRLEDAEADNDPILG

CAA76740 NLLTNPDNHAGLDRGHFLSRTGNCNTFDDGADGYCRADGVGTIVLKRLEDAEADNDPILG

CAA46695 NILTNPDNHAGLDRGHFLSRTGNCNTFDDGADGYCRADGVGTVVLKRLEDALADNDPILG

XP_002147717 NVLTNPDNHAGLDRGHFLSRTGNCNTFDDAADGYCRADGVGTVVLKRLEDAIADNDPIQA

AAC41675 NMIYTPDGHTGLDKGFFLSRTGNCKPYDDKADGYCRAEGVGTVFIKRLEDALADNDPILG

ACS74449 NVLTNPDIFSGLSKGQFLSKTGGCKTYDNDADGYCRGDGCGSVVLKRYEDAIADQDNILG

ACS74442 NVLTNPDIFSGLSKGQFLSKTGSCKTYDNDADGYCRGDGCGSVVLKRYEDAIADQDNILG

ACS74444 NVLTNPDIFSGLSKGQFLSKTGSCKTYDNDADGYCRGDGCGSVVLKRYEDAIADKDNILG

LH1 **NVLTNPDIFSGLSKGQFLSKTGSCKTYDNDADGYCRGDGCGSVVLKRYEDAIADKDNILG**

BAD22832 NVLTNPDIFSGLSKGQFLSKTGSCKTYDNDADGYCRGDGCGSVVLKRYEDAIADKDNILG

AAR90272 NVLTNPDIFSGLSKGQFLSKTGSCKTYDNDADGYCRGDGCGSVVLKRYEDAIADKDNILG

ADB77860 NVLTNPDIFSGLSKGQFLSKTGSRKTYDNDADGYCRGDGCGTVILKRYEDAIADKDNILG

AAY00102 NVLTNPDIFAGLSKGQFLSKTGSCKTYDNAADGYCRGDGCATVVLKRYEDAIADKDNILG

TB1 **NVLTNPDIFAGLSKGQFLSKTGSCKTYDNDADGYCRGDGCATIILKRYEDAVADKDNILG**

AAP68704 NVLTNPDIFSGLSKGQFLSKTGSCKTYDNAADGYCRGDACGSVILKRYSDAIADKDNIMG

ABU63483 NVLTNPDIFSGLSKGQFLSKTGSCKTYDNDADGYCRGDGCGSVILKRYEDAIADKDNILG

CF1 NVLTNPDIFSGLSKGQFLSKTGNCKTYDHDADGYCRGDGCGTVILKRYQDAIRDNDNILG

ACJ24817 NVLTAPDIFAGLSRGQFLSKTGGCKTFDDEADGYCRGDGVGTVILKRLEDAEADNDNILA

PT1 SCMTNSDIFAGLSRGQFLSKTGPCDTFDNGADGYCRGDGCASVVVKRLEDAIAEKDNVLA

CAM35471 SCMTNPDIFSGLSRGQFLSKKGPCATFDNEADGYCRGDGCASVVIKRLEDAQADNDRVLA

BAA18956 SCMTNPDIFAGLSRGQFLSKTGPCATFDNGADGYCRADGCASVIVKRLDDALADKDNVLA

ACN32207 SCMTNPDIFAGLSRGQFLSKTGPCATFDNGADGYCRADGCASVIVKRLDDAIADKDNVLA

EEY14472 SCMTNPDIFSGLSRGQFLSKTGPCATFDNGADGYCRADGCASVIVKRLDDAIADKDNVLA

ABD47522 SCMTNSDIFAGLSRGQFLSKTGPCATFDNDADGYCRGDGCASVIVKRLEDAEADGDNILA

AF151533 SCMTNSDIFSGLSRGQFLSKEHNCNTFDNDADGYCRADGCASVIVKRLDDALADKDNILA

ACN22185 SCMTNSDIFSGLSRGQFLSKNNNCNTFDNDADGYCRADGCASVIVKRLDDAIADKDNILA

SS3 SCMTNSDIFAGLSRGQFLSKKGPCATFDNDADGYCRADGVGTVIVKRLDDALADRDNILA

SS1 SCMTNSDIFAGLSRGQFLSKKGPCATFDNDADGYCRADGVGTVIVKRLDDALADKDNILA

AAP79125 SCMTNSDIFAGLSRGQFLSKTGPCATFDNDADGYCRADGVGTVIVKRLDDALAEKDNVLA

AAP79129 SCMTNSDIFAGLSRGQFLSKTGPCATFDNDADGYCRADGVGTVIVKRLDDAIAEKDNVLA

AAN59953 SCMTNSDIFAGLSRGQFLSKTGPCATFDNDADGYCRADGVGTIIVKRLEDAISDKDNVLA

AAR90248 SCMTNPDIFSGLSRGQFLSKNGPCATFDHDADGYCRADGIGTVIIKRLDYALADKDNVLA

ACJ24813 SVMTNSDIFSGLSRGQFLSKVGPCQTFDNEADGYCRADGIGTVIIKRLQDAEADKDNILA

AAY00058 NVMTNPDIFSGLSKGQFLSKTGPCATFDNDADGYCRGDGVGTVILKRLEDAEADNDNILA

LH2 NVMTNPDIFSGLSKGQFLSKTGSCKTYDDSADGYCRGDGVVTLILKRLDDAVADQDPILG

AAR90249 NVMTNPDIFSGLSKGQFLSKTGSCKTYDDSADGYCRGDGVVTLILKRLDDAVADQDPILG

AAP68703 NVLTNPDIFCGLSRGHFLSPTGSCKTFDDSADGYCRGDGVCTVILKRLDDALADNDPILG

ACJ39412 NVLTNPDIFSGLSRGHFLSKTGQCKTYDNGADGYCRGDGVASIILKRMEDAVADRDPILA

AAN74983 TLFTSADTFCGLSRGHFLNHTGNCKTFDDAADGYCRGEAVATVVVKRLSDAKADNDKVLA

BS1 NVLTNPDIFSGLSKGQFLSKTGSCKTYDESADGYCRGDGVVTLILKRLDDAIAENDPILG

AAD39830 NLILGPMTFVLESKLSMLSPSGRSRMWDAGADGYARGEAVCSVVLKTLSQALRDGDTIEC

BAC20564 NLILGPMTFVMESKLNMLSPNGRSRMWDAAADGYARGEGVCSIVLKTLSQALRDGDSIEC

AAV66106 NLILNPTQYVAESNLRMLSPTGRSRMWDASADGYARGEGIASVVLKTLSQAIADGDSIEC

AAD43562 NLVFSPTMSVAMTEQGVLSPDASCKTFDANANGYARGEAINAIFLKPLNNALREGDPIRA

. . . . .*. :* *:**.*.:. ::.:: . * : * :

ABQ85550 VIAGAYTNHSAEAVSITRPHAGAQAFIFDKLL

CAB44712 IIGGAYTNHSAEAVSITRPHVGAQSFIFDKLL

SS2 IIGGAYTNHSAEAVSITRAACGSTVLHLQAS-

LJ1 IINGAYTNHSAESVSITSPLAGAQSFIFDKLL

ACJ13039 VINAAYTNHSAEAVSITRPHVGAQAFIFNKLL

CAA76740 VINAAYTNHSAEAVSITRPHVGAQAFIFNKFF

CAA46695 VINGAYTNHSAEAVSITRPHVGAQAFIFKKLL

XP_002147717 VIAGAYTNHSAEAVSMTRPHSGAQAFIFDKLL

AAC41675 VILDAKTNHSAMSESMTRPHVGAQIDNMTAAL

ACS74449 CILGAATNHSAEAVSITHPHAGAQEYLYSKVL

ACS74442 CILGAATNHSAEAVSITHPHAGAQEYLYNKVL

ACS74444 CILGAATNHSAEAVSITHPHAGAQEYLYSKVL

LH1 **CILGAATNHSAEAVSITHPHAGAQEYLYNKVL**

BAD22832 CILGAATNHSAEAVSITHPHAGAQEYLYNKVL

AAR90272 CILGAATNHSAEAVSITHPHAGAQEYLYNKVL

ADB77860 CILGAATNHSAEAVSITHPHAGAQEYLYKKCY

AAY00102 CILGAATNHSAEAVSITHPHAGAQEFLYKRVL

TB1 **CILGAATNHSAEAVSITHPHAGAQEFLYKQVL**

AAP68704 CILGAATNHSAEAVSITHPHAGAQEFLYKQVL

ABU63483 CILGAATNHSAEAVSITHPHAGNQEFLFKKVL

CF1 SILGSGTNHSAEAVSITHPHAGAQEYLYNKVL

ACJ24817 TILGTATNHSAEAVSITHPHAGAQEFLYKKVL

PT1 VILGTATNHSADAISITHPHGPTQSILSSAIL

CAM35471 VILGTATNHSADAISITHPHGPTQSTLSQAIL

BAA18956 VILGTATNHSADAISITHPHGPTQSILSRAIL

ACN32207 VILGTATNHSADAISITHPHGPTQSILSRHIL

EEY14472 VILGTATNHSADAISITHPHGPTQSILSSAIL

ABD47522 IILGTATNHSADAISITHPHGPTQSILSSAIL

AF151533 VILGTQTNHSADAISITHPHGPTQSILSSSIL

ACN22185 VVLNAQTNHSADAISITHPHGPTQSVLSASIL

SS3 VILATATNHSADAISITHPHGGTQEILYKSIL

SS1 VILDTATNHSADAISITHPHGGTQEILYKSIL

AAP79125 VILATATNHSADAISITHPHGGTQEILYKSIL

AAP79129 VILATATNHSADAISITHPHGGTQEILYRSIL

AAN59953 VILGSATNHSADAVSITHPHGGTQEILYRSIL

AAR90248 VILGSATNHSADAVSITHPHGGTQEILYKRIL

ACJ24813 VILGSATNHSAEAISITHPHAETQETLYKKIL

AAY00058 VILATATNHSADAVSITHPHDKTQEILYKKVL

LH2 VIAGIATNHSAEAVSITHPHAGAQKFLFQKVM

AAR90249 VIAGIATNHSAEAVSITHPHAGAQKFLFQKVM

AAP68703 VIKGTGTNHSADAVSITHPCAKDQAFLFSKVL

ACJ39412 VIKGTGTNHSADAVSITHPCAKDQAFLFSKVL

AAN74983 VILAAGTNYSAASASITHPHGPTQETLYRRLL

BS1 VIGGIATNHSAEAISITHPHAGAQKFLFQKVM

AAD39830 VIRETGVNQDGRTTGITMPNHSAQEALIKATY

BAC20564 VIRETGINQDGRTTGITMPNHSAQEALIRATY

AAV66106 IIRETGVNQDGRTSGLTVPSNIAQTKLIRETY

AAD43562 LVRATSSNSDGKTPGMSMPSSESHEALIRRAY

: * .. : .:: .

Fungal NRPS multiple sequence alignment

YP_002908545 LLHCYGPTENTTFST-TCEITADDARLPRLPIGRPIANTRVYLLDAHGQPVPLGAAGELH

AAF99707 LLNGYGPTEATTFSA-TYEITSVDN--GSIPIGKPVGNTRLYVLDSQGQPAPLGVAGELY

CAM34312 MINGYGPTENTTFTS-CYTVPSEESTGTSVPIGRPIANTTVYVLGAHHQPMPIGVTGRLY

ACM68684 LLHVYGPTESTTFTS-WYLIKNIPSEATTIPIGRPLANTEIYILDPYLQPVPIGVKGELH

CAQ48266 VFNAYGPTESTVCTT-IALIKD-PQEKP--PIGKPLGNFQVYILDPCLNPVPIGINGELY

ACZ55942 LFNSYGPTEATVVTT----LELLTP-VAPVSIGRPISNAQVYILDQYLQPVPIGVPGEMH

ABW84363 FFNAYGPTETTVCAT----VARFTKDEEKVSIGRPVPNTQIYILDSYLQPVPIGVPGELH

CAQ48260 IINGYGPTESTTFTCCYPIPKQLETKIKSIPLGKPIANTQVYILDKYLQPVPVGVSGELH

PPE2 EFALYGPTESTVCASLIVCKDD----KTSSSIGRPIANTQLYILDGQLQPVPVGVAGELH

PPE1 EFALYGPTESTTFACCYAIKPEDAFATGSVPIGRPIANTQLYILDGQLQPVPVGVAGELH

AAF17281 LHNHYGPSESHVIIT-FTLNNSVETWPLLPPIGRPIANTQIYILDKYLQPVPVDVAGELH

PP3 EFALYGPTETHVASA-FTLPAEVAQWPALPPIGKPIANTRIYLLDSQGQPVPVGVAGELH

CAA72310 LHNHYGPAETHVMTGIELPV-DPGGWPERVPIGGPVDNARLYVLDGFLRPVPPGVVGELY

YP_347997 LHNQYGPTETHVVSQFSLDCNDAESWPDAPPIGRPIANARLYVLDEHLNPVPVGVAGELY

Q70LM5 LANQYGPTENAVVATAGIVP-AAAGQVSAPSIGRPIDNVQVYVLDEKLQPVPIGVAGELY

ABJ99088 LHNRYGPTETAINVTHWQCT-EADGERSP--IGRPLGNVLCRVLDSDLNPVPAGVPGELC

Q70LM4 FFNCYGPTEATVCST----MMLCQAGMNNPPIGRPIANATVYVLDANLNPVPVGVPGELY

CAA82227 VFNAYGPTENTILST-IYNVAENDSFVNGVPIGSAVSNSGAYIMDKNQQLVPAGVMGELV

ABR28366 VYNAYGPTENGVMST-IYKVTGNDSFINGVPIGRAISNSGAYIMDPNQQLVPAGVMGELV

ADB27871 CFNAYGPTENGVFST-MYNVAENESFHNGLPLGRPLNNSGAYVMDPNQQLVGVGVMGELV

Q00869 IANIYGPTEAGIIST-CYNIPKDEAYTNGVPIGGSIYNSGAYVMDPNQQLVGLGVMGELV

AAX09985 VFNAYGPTENSVMST-LYLLSDNEACVNGVPIGRSISNSGAYVMDPEQNLVPLGVVGELV

***:* :* .: * ::. . . *.:

YP_002908545 LGGDGAALGYLGRPDLSAERFLADPFDPTP-----GARLYRTGDLA

AAF99707 IGGQGVARGYLHRDELTLEKFLADPFDSDP-----QARLYRTGDLV

CAM34312 TGGDGVARGYINDAALTADRFIPDPFSGRP-----GARLYDTGDLA

ACM68684 IGGDGLARCYLNRPDLTEQKFIPNPFSQDS-----SARLYKTGDIV

CAQ48266 IGGEGLAKGYLGQPELTNSKFISNPFNDDP-----ASRLYKTGDIV

ACZ55942 IGGAGLARGYWQRPELTHAKFIPNPFEELG-----NSKLYKTGDLA

ABW84363 IGGAGLARSYLNRPELTQEKFIPNLFDKAE-----GSKLYKTGDLA

CAQ48260 IGGAGLARGYLNRLELTAEKFIPNPFEPLSKVSNQQSKLYKTGDLA

PPE2 IGGDGLARGYLNRPELTAEKFIAHPFSDEP-----GARLYKTGDLR

PPE1 IGGDGLARGYLNRPELTAEKFIAHPFSDEP-----GARLYKTGDLR

AAF17281 IGGVSLARGYLNRPELTQQRFIPNPFSTDP-----DSRLYKTGDLA

PP3 IGGAGVARGYLNREELTAQRFLPDPFSGEA-----EARMYKTGDLR

CAA72310 LAGAGVARGYLNRPGLTAERFVADPFGG-PG-----TRMYRTGDLA

YP_347997 IAGACLARGYLNRPDLTAERFLPDPFSAEPG-----ARMYRSGDLA

Q70LM5 IAGDSLARGYLHRPDLTRERFIANPYGQKAG-----ARMYKTGDLV

ABJ99088 IGGLGLARGYLGRPGLSAERFVADPLGP-AG-----ARLYRTGDRA

Q70LM4 IGGKGLARGYWNRPELTAESFIPHPFGTAG------ERLYRTGDLV

CAA82227 VTGDGLARGYMD-PKLDADRFIQLTVNGSEQ-----VRAYRTGDRV

ABR28366 VTGDGLARGYTD-STLDIDRFVQVNIDG-QL-----VTAYRTGDRV

ADB27871 VTGDGLARGYSD-PTLDKNRFIHINMDG-ET-----VRAYRTGDRV

Q00869 VTGDGVGRGYTN-PELNKNRFIDITIEG-KT-----FKAYRTGDRM

AAX09985 VIGDGVARGYTD-PNRNVDRFVTITVGN-QT-----MRAYRTGDYV

* . * *: * :**

Bacterial PKS multiple sequence alignment

ABI94380 LGSLKSNIGHAQAAAGVGGVIKMVMALRHGVLPRTLHVDEPSTQVDWTQGDVRLLTDAVP

DL1 IRPFKSNIGHTQAAAGVAGIIKMVLALQHGLLPRTLNVDAPSSHVDWSVGAVELLAEAQP

ABJ97437 LGSVKSNIGHTQAAAGVAGVIKMVMAMRHGVLPQTLHVDEWSPHVDWSGGRVELLTEATP

YP_882292 LGSIKSNIGHTSAAAGVAGVIKMVQALRHGVMPKTMHVDVPSPHVDWSAGAVSLLTDPRP

NP_733695 LGSLKSNIGHAQAAAGVGGVIKMVEAIRHGVLPRTLHVDEPSPRVDWNAGALELLTGERA

ABV83221 LGSVKSNIGHTQSAAGVASVIKTVMALRHGVLPRTLHADEPSSHVDWSAGSVELLAEQTA

AAP45193 LGSVKSNLGHTQAAAGVAGVIKMVEALRHGVMPATLHADTPSSQVDWSAGAVELLTEARD

AAK73514 LGSLKSNIGHTQAAAGVAGVIKMVMSMRHGVLPCTLHVDAPSSHVDWTEGAVELLTEQTE

AAF71776 LGSLKSNIGHTQAAAGVAGVIKMVMAMRHGVLPQTLHVDAPSSHVDWSVGAVELLTEQTA

ABB86421 LGSLKSNLGHTQAAAGVASVIKTVEALRHGVMPATLHADTPSSQVDWSAGAVELLTEARD

ABB86422 LGSLKSNLGHAQAAAGVAGVIKMVEALRHGVMPATLHADTPSSQVDWSAGAVELLTEARD

CAA11038 LGSVKSNLGHTQAAAGVASVIKMVQALRHGVLPPTLHVDRPSTEVDWSAGAVSLLTEARE

CAA11039 LGSVKSNFGHTQSAAGVAGVIKMVQALRHGVMPPTLHVDRPTSQVDWSAGAVEVLTEARE

AAS46347 LGSIKSNVGHTQYAAGVSGVIKTVMALRHGVMPKTLHVDEPTPHVDWSSGAVRLLTEARE

AAY28226 LGSIKSNIGHTQAAAGVAGVIKMVQAMRHGVLPKTLHADEPTTKVDWSQGAVSLLSEARP

ABB86409 LGSIKSNIGHTQAAAGVAGVIKMVQAMRHGVLPKTLHADEPTSKVDWTSGAVSLLSEARP

BAD08360 LGSAKSNFGHTQAAAGVAGVIKMVMAIRNGVLPKTLHVSEPSTHVDWSAGAVELLAEARE

ZP_06506124 VGSIKSNMGHTQAAAGVAGVIKMVQAMRHGVMPATLHVDEPSPRVDWTSGAVSVLTEARE

Ppol1 IRPLKSNIGHTQAAAGVAGVIKMVQALRHGVMPPTLHVDVPSTKVDWTAGAVELLTEPRK

Ppol2 IRPLKSNIGHTQAAAGVSGVIKMVEALRHGVMPPTFHVEEPTPQVDWTAGAVELLREARK

AAF62885 LGAVKTNLGHLEGAAGVAGLIKAALALHHESIPRNLHFHTLNPRIRIEGTALALATEPVP

AAF26919 IGSVKTNLGHPEYASGITGLLKVVLSLQHGQIPAHLHAQALNPRISWGDLRLTVTRARTP

AAF00958 VASVKTNIGHLEAAAGMAGIIKTILILQQGEIPPHLHFQSPNPLINWQDHPIEIPTQNIP

AAK57187 IGSVKTTIGHLEAAAGIAGLIKVVLAHRHGVVPPHLHLKKLNPHIELNGFPLDIPTQVTP

: . *:..** . *:*: .::* :: :* :: .. : : :

ABI94380 WPETGRPRRAGVSSFGVSGTNAH-VILEAPEATGP--EPGPEP----GSEVGGPVPWVLS

DL1 WPTNGRVRRAGVSSFGVSGTNAHLIIVEAPTALTAPVAPGSTAPVGSGPTVGESVPWVLS

ABJ97437 WPMT--VRRAGVSSFGVSGTNAHIILEQAPETAQP---TRPIP---EGDTEVAAVAWVLS

YP_882292 WPEHGGPRRAGVSSFGISGTNAHVIVEQAPAAAETEAAPASSTMP------DAVVPWVVS

NP_733695 WPDTGRPRRAAVSSFGISGTNAHVVLEQAPDAP-PPSAAPPATQAPAVP-----APWLLS

ABV83221 WPETGRPRRAAVSSFGISGTNAHTVLEQAP--EAAPVAPATPSGPVLPSAPEPVLPWVLG

AAP45193 WPDTGRPRRAAVSSFSMSGTNAHLILEHSPE--------PQAESVAEPV--SDVVPLVVS

AAK73514 WPETDRVRRAAVSSFGISGTNAHVILEQPVVAP-APATADVDGAEPAV------VPLILS

AAF71776 WPETGRVRRAGVSSFGISGTNAHVIVEQPALVE-SPA-AEPSGREPGV------VPLPLS

ABB86421 WPDTGRPRRAAVSSFSMSGTNAHLILEHSPE--------PQAESVSEPV--SDVVPLVVS

ABB86422 WPDTGRPRRAAVSSFGVSGTNAHLILEQAPVEQSAVPVPGESG-LVGTD--GGVVPLVLS

CAA11038 WPREGRPRRAGVSSFGISGTNAHLILEEAPE--------EEPPVAEAPS--AGVVPVVVS

CAA11039 WPRNGRPRRAGVSSFGISGTNAHLIIEEAPA--------EPQLAGPPPD--GGVVPLVVS

AAS46347 WPETGRPRRAGVSSFGVSGTNAHVILEQAPEAEP----VEVDEADRPVL--MGSVPWVVS

AAY28226 WPETGHPRRAGISSFGVSGTNAHVILEQAPPEVAVPEAEASETGTPGLVATGGVVPWMLS

ABB86409 WPETGHPRRAGISSFGVSGTNAHVVLEQAPLEAAAPEVDVDEAGAPGLVATGGVVPWVLS

BAD08360 WPETGRPRRAGVSSFGVSGTNAHVIVEQAPTEEQAPADAPAPTDAPAGTP----VPWIVS

ZP_06506124 WSVDGRPRRAAVSSFGISGTNAHLILEEAPVPAPAEAPVEASESTGGRGPRPSMVPWVIS

Ppol1 WPDTGRPRRAGVSSFGVSGTNAHLILEQAPEEQPVTPVESVSESMVDVV--DGVVPLVLS

Ppol2 WPDTGRPRRAAVSSFGASGTNAHVILEESPE-KPVEAPAAESGVELPAA--PVVVPLVVS

AAF62885 WPRAGRPRFAGVSAFGLSGTNVHVVLEEAPATVLAPATPGRS-----------AELLVLS

AAF26919 WPDWNTPRRAGVSSFGMSGTNAHVVLEEAPAATCTPPAPERP-----------AELLVLS

AAF00958 WPNNNKVPIAGVSSFGFSGTNAHVIVQQAPVSKISEIQQQIP-----------SHLLTLS

AAK57187 WPERARPRIAGISAFGLSGTNAHVLVEEAPAPEPRAEQAERS-----------LHVLALS

*. *.:*:*. ****.* :: .. :.

ABI94380 ARTAEAVRAQAARLVEHVEADPSLRPVDVGWSLVDTRAVFDHRAVVVGSDREQLLAGVR-

DL1 ARNETALREQALRLAAFVVEHPELDPVTVGRALATTRAVFGHRAVVVASTREDFVSALHG

ABJ97437 GQGEAGLRAQAERLRAFTAASPCPTPAEVGWSLASTRTTLSHRAVVVGAGRDELLRGVDA

YP_882292 ARSAEALAGQARRLLDHVTADAQASPLDVGWSLVSTRAVFEHRAVVVGRERGALATGLAG

NP_733695 ARDPDTLRAQAERLRAYADGDRAPEPAAIGRALATTRTAFEQRAVVLAEDRAGYLAGLDA

ABV83221 ARGAAALRGQADRLLAHLET-TDARPADIGFSLVSARMPFEHRAVVVATDPADRAAGLRA

AAP45193 ARSARSLVGQAQRLASFVGSASDVALPEVAAGLVSRRAVLSERAVVVAGSRDQALSGLGA

AAK73514 GKSPEALRDQAARLLDTVREHTALRPLDLGHSLATSRSAFDHRAVVLATGREDALRALTA

AAF71776 GKSPEALRDQAARLLAGLAERPALRPLDLGYSLATTRSAFDHRAVVLATDRADAVRALTA

ABB86421 ARSARSLVGQARRLASFVGSQSDVVLPEVAAALVSRRAVLSERAVVVAGSRDEALSGLGA

ABB86422 ARGAAGLAGQADRLGSFLSAHTDLDLAEVARSLVSTRGALSDRAVIVAGSRDEALTSLDA

CAA11038 AR--GALAGQAGRLAAFLEA-SDEPLVTVAGALICGRSRFGDRAVVVAGTRAEATAGLAA

CAA11039 ARSPGALAGQARRLATFLG---DGPLSDVAGALTS-RALFGERAVVVADSAEEARAGLGA

AAS46347 ARGEGALRAQAGRLLEWLVERPGLGPVDVGFSLVGTRSAFEQRAVVLGGDREELLAGLRS

AAY28226 GKTPAALRAQAERLVSHLESGSDANPVDVGWSLATTRAALDHRAVILATDTEDGIATARA

ABB86409 AKTPAALRAQAERLVSHLESGDAPNAVDVGWSLATTRAALEHRAVILATDTEGGMATARA

BAD08360 GRTADSLRDQARRLLEHLDRNGDLDPQDVARALLTTRTRFHHRAAVVATERQDIVAALEA

ZP_06506124 ARSAEALTAQAGRLMAHVQANPGLDPIDVGCSLAS-RSVFEHRAVVVGASREQLIAGLAG

Ppol1 AKSAVSLAGQAERLASFVEAGEEVLLPEAAAALVARRAVLSERAVVVAGSRGEALAGLEA

Ppol2 ARSPGSLVGQAERLVSFVGENAGT-LPQVAGALVARRALLSERAVVVAGSGEEALAGLGA

AAF62885 AKSTAALDAQAARLSAHIAAYPEQGLGDVAFSLVATRSPMEHRLAVAATSREALRSALEA

AAF26919 ARTASALDAQAARLRDHLETYPSQCLGDVAFSLATTRSAMEHRLAVAATSREGLRAALDA

AAF00958 AHNKTALKELAKRFHTLLESHP--EIGDICYSAAVGRIDLPERLAIVGDTCPELQQRLAA

AAK57187 AKTKGALTQLAARVGEYLAEQPEQKLADVCHAANTQRTWFSHRLALVVESVEQTRERLLA

.: : * *. . * : .* .:

ABI94380 ------HVSPVAAG----ADPR--VGVLFTGQGAQRVGMARGL

DL1 WQLAPQVVQGVAAG----VGSG--PVWVFPGQGAQWIGDGSRF

ABJ97437 VANG-MAAPGVVRG----TGAPGDVVFVFPGQGSQWVGMALEL

YP_882292 LASG-RPGPATVVG---RARSTGKTVLVFPGQGSQTLGMGRQL

NP_733695 LIRG-ADAPGVVTG---SARSAGRTAFLFTGQGAQRAGMGREL

ABV83221 VAAD-GPSGVVARG---VADVEGRTVFVFPGQGSQWLGMGARL

AAP45193 LARG-ESHFGVVTGGSLGGAVSGRKVLVFPGQGSPWVGMGREL

AAK73514 LADD-EANSAAVTG---RTR-SGRRAALFSGQGSQRLGMGREL

AAF71776 LAAA-DADLSAVVG---DTR-TGRHAVLFSGQGSQRLGMGREL

ABB86421 LAGD-ESHPGVVTGGSLGAAVSGRKVLVFPGQGSPWVGMGREL

ABB86422 LALG-HSAAGVVTGSARDPNAAGRVVFVFPGQGAQWAGMGADL

CAA11038 LARG-ESAADVVTGTVAASGVPGKLVWVFPGQGSQWVGMGREL

CAA11039 LARG-EDAPGLVRGRVPASGLPGKLVWVFPGQGTQWVGMGREL

AAS46347 VAEG-VPGAGVVSGRAAGDGGMG-VVFVFPGQGSQWVGMGREL

AAY28226 LAEG-RPDPLLVTG---QTGTDGKTVFVFPGQGAQWVGMGAQL

ABB86409 LAEG-RPDPLLVTG---QTGTDGKTVFIFPGQGAQWVGMGAQL

BAD08360 LADG-RPVSGLVQG---TATTMAKSAFLFTGQGAQRVGMGREL

ZP_06506124 LAAG-EPGAGVAVG---QPGSVGKTVVVFPGQGAQRIGMGREL

Ppol1 LARG-ESHPGVVTGGLSSAVVAGRTVLVFPGQGSQWAGMGREL

Ppol2 LARG-ESCPGLVIG---SAVVAGRTVLVFPGQGLQWAGMGREL

AAF62885 AAQG-QTPAGAARGR--AASSPGKLAFLFAGQGAQVPGMGRGL

AAF26919 AAQG-QTSPGAVRSI--ADSSRGKLAFLFTGQGAQTLGMGRGL

AAF00958 FAEE-NPLDDLTFYQRFTSEKSPKIVFLFTGQGACYPGMGHQL

AAK57187 FARG-EEVPRLAQNR--VEGEDPKVVFVFTGQGAQYAGMGREL

. :*.*** * . :

Bacterial NRPS multiple sequence alignment

AAG34184 LAYVIYTSGS--TGRPKGVAV-THAGLANLVAAKVERMDVDE--------

AAM47273 PAYVIYTSGS--TGRPKGVVV-THAGISTFSAAEVAHLDVRP--------

BC2 LAYVIYTSGS--TGKPKGVIV-THLGLSNLNAEEHQRFNVQP--------

BC1 LAYVMYTSGS--TGKPKGVIV-THLGLSNLNAEEHQRFNVQP--------

ZP04387369 PAYLIYTSGS--TGKPKGVIV-THLGLSNLNAEEHQRFNVQP--------

YP_705042 PAYLIYTSGS--TGRPKGVTV-THRGMADFTAETHQRFQVTH--------

YP_002782356 PAYLIYTSGS--TGRPKGVTV-THRGMADFTAETHERFQVSH--------

ABD65957 PAYVIYTSGS--TGRPKGVVV-THEGCANLSAS-HDWYGVAA--------

CAA72310 PAYVIYTSGS--TGLPKGVPV-PHRSVASVLVPLIEEFGLGP--------

YP_001063288 LAYVIYTSGS--SGEPKGVMN-EHRGVVNRLWWMQQTYALDE--------

ABN87372 LAYVIYTSGS--SGEPKGVMN-EHRGVVNRLWWMQQTYALDE--------

ABH06368 AAYVIYTSGS--TGQPKGVIN-EHAGVVNRLLWMQDAYGLKA--------

BAC67535 TAYVIYTSGS--TGQPKGVIN-EHSGVVNRLLWMQDAYQLTS--------

ABW17377 LAYVIYTSGS--TGLPKGVMN-EHAGVVNRLLWMQDAYNLGA--------

ABW17376 LAYVIYTSGS--TGLPKGVMN-EHAGVVNRLLWMQDAYNLGA--------

ZP_06710581 PAYVIYTSGS--TGRPKGVVV-PHSAIDNRLRWMQHAYGLTG--------

DL2 LAYVMYTSGS--SGRPKGVS---RGALANRLWWAQETFGLTS--------

CAJ14037 LAYLIYTSGS--TGRPKGVLN-EHGPVCNRIRWGMPAFPPGP--------

YP972054 LAYVIYTSGS--TGRPKGVAV-RHAALHTCMAWMQGTYGLAA--------

LH1 LAYPIYTSGS--TGRPKGAAV-RHRSLAGCMRWMQDTYGLTR--------

AAF40219 LAYVIYTYGS--TGQPKGVGN-THGALAERLQCMQTTYQLDE--------

LH2 LAYVMYTSGS--TGQPKGVGN-THVALAERLQWMQNAYRLND--------

ABU63483 VAFCFTGQGSQYTGLGQKLYQ-DLPSFKAD----IDQLNQLAESHNLPSF

AAN59953 VAYTFTGQGSHYAGLGKDLYN-NSSVFREN----ILEFDRIARIQGFPSF

AAF26919 LAFLFTGQGAQTLGMGRGLYD-VWSAFREAFDLCVRLFNQEL--DRPLRE

AAF71776 HAVLFSGQGSQRLGMGRELYE-RFPVFAEALDVAIDHLDAALPAQASLRE

DL3 LAYVMYTSGT--AGSPKAVVSTQRAALWSPLACYGPMLGLSA--------

* : *: * :

AAG34184 ---------------QSRILQFASPSFDAFMTELLATIGAGATLVVPPPG

AAM47273 ---------------GDRVLEFSSPSFDASVLELCMALPAGAALVVPPPG

BC2 ---------------YSRISHLASPSFDASVFELMMAFGSGACLVVIPPT

BC1 ---------------YSRISHLASPSFDASVFELMMAFGSGACLAVIPPT

ZP04387369 ---------------YSRISHLASPSFDASVFELMMAFGSGACLVVIPPT

YP_705042 ---------------ESRVSQLASPSFDASVFELMMAFSASARVVIVPPA

YP_002782356 ---------------ESRVSQLASPSFDASVFELMMAFSASAQVVIVPPA

ABD65957 ---------------GSRVAQFASVGFDMFCEEWLLALLRGATLVTVPAD

CAA72310 ---------------GSRVLQFASISFDAALWEITLALLSGATLVVAPAE

YP_001063288 ---------------RDAVLQKTPFSFDVSVWEFFWPLMSGARLVIAKPE

ABN87372 ---------------RDAVLQKTPFSFDVSVWEFFWPLMSGARLVIAKPE

ABH06368 ---------------HDAVLQKTPFSFDVSVWEFFWPLFTGARLVMARPE

BAC67535 ---------------SDTVLQKTPFSFDVSVWEFFWPLMTGARLVMARPE

ABW17377 ---------------EDVVLQKTPFSFDVSVWEFLWPLQTGACLVMARPG

ABW17376 ---------------EDVVLQKTPFSFDVSVWEFLWPLQTGACLVMARPG

ZP_06710581 ---------------EDRVLQKTPSSFDVSVWEFFWPLREGATLVVAEPG

DL2 ---------------ADRVLVKTPFSFDVSVWELLWPLMVGSSVVVARPG

CAJ14037 ---------------GTIVLQKTPIHFDVSVWEMFWTLATGATLVLARPD

YP972054 ---------------PDTVLHKAPFGFDVSCWEIFWPLTAGARLLVAPPG

LH1 ---------------DDTVLHKAPFGFDVSVWEIFWPLTTGVRLVVANPG

AAF40219 ---------------SDVLMQKAPISFDVSVWECFWPLITGCRLVLAGPG

LH2 ---------------TDVLMQKAPISFDVSVWECFWPLITGARLLIAGPG

ABU63483 LELFDG--TDVATLSP-VKVQLGTACIQVALSRMWESWGIKPSAVIGHSL

AAN59953 LPLIDGSVTDVSTLSP-VVVQIGMVCFEVAMARLWASWGVNPSVLVGHSL

AAF26919 VMWAEPASVDAALLDQTAFTQPALFTFEYALAALWRSWGVEPELVAGHSI

AAF71776 VMWGD----DVELLDETGWTQPALFAVEVALFRLVESWGVRPDFVAGHSI

DL3 ---------------DDRVLWPLP--MAHSFAHSFCVLGVVAAGASARIC

.

AAG34184 ILA-GDHLAEVLVAERITHVVLPPVAAASVSPESLPD-----LRSLVLAG

AAM47273 PLL-GDQLADVVDEFGVTHALIPPVALATVPDRPLPT-----FRTLVVGG

BC2 VFG-GSEWAEIFADEHVSHAFITPTALSSIESSALPE-----LRVLAVGG

BC1 VFG-GSELAEIFADEHVSHAFITPTALSSIESSALPE-----LRVLAVGG

ZP04387369 VFG-GSELAEIFADEHVSHAFITPTALSSIESSALPE-----LRVLAVGG

YP_705042 IVG-GSELADLFRREQVTHATITPTALAALDNDGLDS-----LRVLDLVG

YP_002782356 VLG-GSELADLFRREQVTHATITPTALAALDNDGLDA-----LRVLDLVG

ABD65957 RRL-GPDLGHFLVDQGVTHAALPPAVAATIPDGLLDP-----SFVLDVGG

CAA72310 QLQPGPALAELVARTGTTFLTLPPTALAVLADDALPA-----GVDLVVAG

YP_001063288 GHKDPAYLSELIDRERVTTLHFVPSMLQAFLEDEGAARGCGSVKRVMCSG

ABN87372 GHKDPAYLSELIDRERVTTLHFVPSMLQAFLEDEGAARGCGSVKRVMCSG

ABH06368 GHKDPAYLCEVIAAEHITTLHFVPSMLDVFLAHGDISQ-AVGLVRVMCSG

BAC67535 GHKDPQYLSEIIEREQITTLHFVPSMLDVFLAHADTAR-CSSLRQVMCSG

ABW17377 GHRDPEYLRQVIRSEGVTTLHFVPSMLDVFLAHGDAS--ADALKRVLCSG

ABW17376 GHRDPEYLRQVIRSEGVTTLHFVPSMLDVFLAHGDAS--ADALKRVLCSG

ZP_06710581 VHKDPAQLARLIHEQAVTTCHFVPSMLQVFLAEPAAAHCAGVLRRVFCSG

DL2 GHRDPAYLAG----LGASTVHFVPSMLEAFLD-AGVRPVG--LRRVLCSG

CAJ14037 GHRDPQYLAGRLVEEGVTDVHFVPSMLAAFLD-VGALPEGHSLRRVFCSG

YP972054 AHRDPERIVQLIERHQVTTLNFVPSMLRAFLDHPGIEH-RTRLRHVICGG

LH1 DHRDPERITALVRRHAVTTMNFVPPMLQAFLAHEGIER-ETRLRYVICGG

AAF40219 EHRDPHRIAQLVQEHGVTTLHFVPPLLQLFVDEPLAAK-CTSLRRVFSGG

LH2 EHRDPHRIAQLVQEYGVTTLHFVPPLLSLFIDEPLSAE-CTSLRRVFSGG

ABU63483 GEYAALHVAGVISASDMVYLVGRRAELLVKDCTPHTHGMLAVKASVDAIR

AAN59953 GEYSALCVSGVLSASDTIYLVGARAQLLVEKCTANTHAMLAVQGSVDALN

AAF26919 GELVAACVAGVFSLEDAVFLVAARGRLMQALPAG--GAMVSIEAPEADVA

AAF71776 GEIAAAHVVGVFSLEDACRLVAARATLMQALPTG--GAMIAIQAAEDEVT

DL3 ERRDPAWLAHLIGTCSPTVVAGVPATYRQLLAAGVGEIAS--LRLCLTAG

AAG34184 EASSGDLIARWAP---GRRVINAYGPTEATVCATMS--EP----LSADAT

AAM47273 DACSADLVARWAP---GRRMVNAYGPTESTVVTSWS--RP----LAPGGV

BC2 EACPPELVDIWGR---NRRMFNGYGPTESTIQASVS--EP----MRPGKD

BC1 EACPPELVDIWGR---DRRMFNGYGPTESTIQASVS--EP----MRPGKD

ZP04387369 EACPPELVDIWGR---DRRMFNGYGPTESTIQASVS--EP----MRPGKD

YP_705042 EACPPEVVARWAP---GRSLHSGYGPTETTIQASVS--DP----MRPGES

YP_002782356 EACPPEVVARWAP---GRSLHSGYGPTETTIQASVS--AP----MRPGES

ABD65957 EACPPELVERWTAD--GRTMFNAYGPTEATVDATVWRCAPG---LDAGAA

CAA72310 EATSPDQVGRWST---GRRMTNAYGPTEAAVCTTIS--AP----LTGAVV

YP_001063288 EALPPSLVKRFYRCLPDARLHNLYGPTEAAVDVTAWACDAE----EGGAS

ABN87372 EALPPSLVKRFYRCLPDARLHNLYGPTEAAVDVTAWACDAE----EGGAS

ABH06368 EALPGSLVRRFKQQLPGIGLYNLYGPTEAAVDVTAWNCARP----EVPDN

BAC67535 EALPGSVVRRFKQQLPASQLHNLYGPTEAAVDVTAWNCAGPL--EQTPDN

ABW17377 EALPGSLVRRFHAQLPTVELHNLYGPTEAAVDVSAWHCVTA------PDN

ABW17376 EALPGSLVRRFHAQLPTVELHNLYGPTEAAVDVSAWHCMTA------PDN

ZP_06710581 EALPRESAHVFARTLPGVELHNLYGPTEAAVDVTYHACDPAD-----SGP

DL2 EALSPGLVDRFFGAFPGVELFNLYGPTEAAIDVSWHRCEPG------ASV

CAJ14037 EALSPGLRDRLFARLPHVELHNLYGPTEAAIEVTHWRCRPG------EPT

YP972054 EAMPETLQREALRRLPGATLQNLYGPTETTIHVTRWTCRDE-----AGP-

LH1 EAMPSATQREALQRLHGVSLQNLYGPTETTIHVTQWTCRDD-----GRSR

AAF40219 KALPAELRNRVLAQWPAVQLHNRYGPTETAINVTHWHCQGA-----DGER

LH2 EALPAELRNRVLAHLPAVQLHNRYGPTETAINVTHWHCSVA-----DGER

ABU63483 NALGSKMT--EVACINGPEETVLCGSSEIVTAANEVLTSKGMKSTKLNVP

AAN59953 EALPEMAGSLSVACINGPRETVLGGEAGAMADLAEKLTQSGFKCTKLQVP

AAF26919 AAVAPHAASVSIAAVNAPDQVVIAGAGQPVHAIAAAMAARGARTKALHVS

AAF71776 QHLT---DDVSIAAVNGPTSVVVSGAESAARTVADRLAENGRKTTRLRVS

DL3 APSDPQLRADVETAL-GVPLHDCYGSTETCGMVSVEPVGTP----RVAGT

*

AAG34184 PPIGGPIPGAACYVLDEALR---------PVPAGVPGELYLGGAGLARGY

AAM47273 PPIGRPLPGTRVHVLDAEGR---------PVPIGVPGELFVAGVGLARGY

BC2 INIGRPAIGFAGLVLDGHLK---------PVPVGVIGELYVTGPGLARGY

BC1 INIGRPAIGFAGLVLDGHLK---------PVPVGVIGELYVTGPGLARGY

ZP04387369 INIGRPAIGFAGLVLDGHLK---------PVPVGVIGELYVTGPGLARGY

YP_705042 VNIGAPARGFAFLVLDERLQ---------PVPVGVPGELYIAGPGMARGY

YP_002782356 VNIGSPALGFAFLVLDERLQ---------PVPAGVPGELYIAGPGTARGY

ABD65957 VPIGRPVLNTRAYVLDDALR---------PVPVGVVGELHLAGSGLARGY

CAA72310 PPIGRPVPNARAYVLDALLQ---------PVPPGVVGELYLAGGGLARGY

YP_001063288 VPIGRPIANTRIYVLDGYGQ---------PVPRGVAGELYIGGVQVARGY

ABN87372 VPIGRPIANTRIYVLDGYGQ---------PVPRGVAGELYIGGVQVARGY

ABH06368 TPIGKPIANTRLYVLDGQLQ---------PVPLGVAGELFIAGVQVARGY

BAC67535 TPIGKPIANTRMYILDAQQQ---------PVPHGVVGELYIGGVQVARGY

ABW17377 TPIGKPIANTTLYVLDTLGQ---------PVPQGVAGELFIGGVQVARGY

ABW17376 TPIGKPIANTTLYVLDTLGQ---------PVPQGVAGELFIGGVQVARGY

ZP_06710581 VPIGRPVWNTRLYVLDAARR---------PCPPGVPGELFLAGRQLADGY

DL2n VPIGRPVANTRLEVWDAGRE---------RVPVEVAGELRIGGVQVADGY

CAJ14037 VPIGRPIANARCYVLDAELN---------PVPPGVPGELWLGGVPVARGY

YP972054 VPIGRPISETQAWVLDAQLQ---------PVPRGVAGELYIGGALLARGY

LH1 VPIGRPIARTQAYVLDAALN---------PVPAGVVGELYIGGELLARGY

AAF40219 SPIGRPLGNVICRVLDAQFN---------LLPAGVPGELCIGGIGLARGY

LH2 SPIGRPLGNVVCRVLDSNLN---------PVPAGVPGELCISGIGLARGY

ABU63483 FAFHSAQVDPILESFRTTASSVSFKKPAVPVLSPLSGDIITDVGVIGPEY

AAN59953 FAFHTAQVDAILDDFEKLAASVRFGSANIPLISPLLGRPLSEGESIDPTY

AAF26919 HAFHSPLMAPMLEAFGRVAESVSYRRPSIVLVSNLSG-KACTDEVSSPGY

AAF71776 HAFHSPLMDPMLAEFRAVAEGLSYATPTLPVVSNLTGRLATADDLCSAEY

DL3 SGLVVPCVEVRVVDPRSALD----------VRDGTDGEIWVRGPGLMSGY

: . * *

AAG34184 LGRPGMTAERFVANPFAG-DG------SRMYRTGDLV

AAM47273 LDRPGLTAARFVADPFGP-PG------SRMYRTGDVV

BC2 HNRPDLTADRFVADPFGE-PG------QRMYRTGDV-

BC1 HNRPDLTADRFVADPFGE-PG------QRMYRTGDV-

ZP04387369 HNRPDLTADRFVADPFGE-PG------QRMYRTGDLV

YP_705042 HNRAALTSERFVACNFGE-PG------CRMYRTGDVV

YP_002782356 HNRPDLTSERFVACNFGE-PG------CRMYRTGDVV

ABD65957 LGRTGLTAERFVACPFQ--PG------RRMYRTGDRV

CAA72310 RNRPGLTAERFVADPFGT-PG------ARMYRTGDLA

YP_001063288 LNRPELTRERFVDDPFVAG--------GRLYKTGDLA

ABN87372 LNRPELTRERFVDDPFVAG--------GRLYKTGDLA

ABH06368 LNRPELTAERFLDDPFTP---------GRMYRTGDLG

BAC67535 LNRPELNAERFLNDPFQAN--------GRMYRTGDVA

ABW17377 LNRAELTAERFIDDPFSTRPG------ARLYRTGDLA

ABW17376 LNRAELTAERFIDDPFSTRPG------ARLYRTGDLA

ZP_06710581 LHRPELTAERFVLDPFGPP-G------SRMYRTGDVA

DL2 VGEPALSAERFVDG---------------WYRTGDVK

CAJ14037 HGRADLTAERFLPDPYGPA-G------SRMYRSGDLA

YP972054 LGQPGLTAERFVADPRG--AG------GRLYRTGDWV

LH1 LGRPSLSAERFVADPVGQ-AG------GRLYRTGDVK

AAF40219 LGRAGLTAERFVADPLGA-AG------ARLYRTGDRV

LH2 LGRPALTAERFVVDPLGE-QG------VRLYRTGRRQ

ABU63483 LAKHARETVNFSQALESGQKAKIFDGKTAWLEIGAHP

AAN59953 LRNHAREAVNFLAGLTSSQELGIIDEKTVWIEVGPHS

AAF26919 WVRHAREVVRFADGVKALHAA----GAGTFVEVGPKS

AAF71776 WARHVREAVRFADGVSTLENE----GVTTFLELGPDG

DL3 HGRSEETRAAMPSG---------------WYRTGDVK

. : . *
